# Plant diversity reduces satellite-observed phenological variability in wetlands at a national scale

**DOI:** 10.1126/sciadv.abl8214

**Published:** 2022-07-22

**Authors:** Iryna Dronova, Sophie Taddeo, Kendall Harris

**Affiliations:** ^1^ Department of Environmental Science, Policy, and Management, Rausser College of Natural Resources, University of California Berkeley, Berkeley, CA 94720, USA.; ^2^ Department of Landscape Architecture and Environmental Planning, College of Environmental Design, University of California Berkeley, Berkeley, CA 94720, USA.; ^3^ Negaunee Institute for Plant Conservation Science and Action, Chicago Botanic Garden, Glencoe, IL 60022, USA.; ^4^ San Francisco Estuary Institute, Richmond, CA 94804, USA.

## Abstract

Plant diversity may enhance stability of ecosystem function and its satellite-derived indicators. However, its potential to stabilize phenology, or seasonal changes in plant function, is little understood, especially in understudied systems with high biodiversity potential such as wetlands. Using a large sample of U.S. wetlands and a new satellite-based indicator of phenological stability, we found that plant diversity was negatively associated with interannual phenological variability after controlling for covariates representing climate, site conditions, and spectral fluctuations. Furthermore, plant diversity and covariates better explained phenological variability than stability in annually summarized satellite-based biomass indicators used by earlier studies. Last, a subsequent path analysis indicated that phenological variability could mediate plant diversity relationship with the latter stability. Our results suggest that contributions of plant diversity to seasonality of ecosystems may have a stabilizing role in their functioning and offer a new basis for assessing biodiversity-stability relationships across broad geographic extents.

## Teaser

Wetland plant diversity stabilizes satellite-based indicators of biomass and the timing of ecosystem functions.

## INTRODUCTION

Biodiversity contributions to long-term stability of ecosystem functioning (*1*–*3*) are critical for halting losses of nature’s benefits to humanity (*4*–*10*). Local-scale experimental studies have attributed these contributions to species asynchrony, response diversity, and functional redundancy (*4*, *11*), which provide an ecological “insurance” against disturbance and stressors (*3*). However, diversity-stability relationships are less well understood in real-world landscapes and at broad geographic scales relevant to species dispersal, management of ecosystem services, and modeling of climate and land use changes (*8*, *10*, *12*, *13*). Furthermore, much uncertainty exists about biodiversity effects on phenology, i.e., timing of seasonal changes in ecosystem function, which can be susceptible to shifts in global and local climates (*12*, *14*–*18*).

Recent efforts to fill these knowledge gaps have increasingly used remote sensing datasets, providing repeated observations over larger geographic extents than possible with field studies alone (*19*). For example, satellite-derived indicators of vegetation biomass, productivity, and their long-term stability have shown positive correlations with plant diversity in terrestrial ecosystems (*5*, *12*, *14*). However, popular indicators of stability, such as the ratios of multiyear mean to variance in annual biomass production or its remotely sensed surrogates such as greenness indices (*2*, *5*, *20*, *21*), do not directly represent phenological variation (*22*). Some studies suggest that biodiversity’s effect on seasonality of ecosystem functions may contribute to their enhancement (*12*), such as longer growing season in diverse and stable plant communities due to phenological asynchronies, adaptation to different environmental conditions within the growing season, and temporal niche differentiation among their species (*12*, *23*, *24*). This raises the question whether biodiversity-phenology linkages play an indirect stabilizing role in addition to the direct effect of diversity on biomass and productivity.

Understanding biodiversity’s role in phenological and functional stability of real-world ecosystems also requires a deeper knowledge of other contributing factors (*4*, *5*, *14*), which themselves can be context dependent (*8*, *20*, *25*). Climate conditions are especially important because interannual fluctuations in temperature and precipitation mediate vegetation phenology and biomass production (*4*, *5*, *12*, *15*, *26*). Remote sensing–derived indicators of vegetation may be confounded by scale-sensitive variation in background reflectance from nonvegetated elements within units of analysis (e.g., pixels or grid cells), obstruction of lower canopy layers by taller vegetation, and environmental fluctuations such as flooding (*22*, *27*, *28*). These and other factors including disturbance and stress may influence the interpretation of biodiversity effects in statistical models (*4*); however, the guidance on them remains limited (*7*, *8*, *20*).

These gaps are especially glaring in complex ecotonal systems such as wetlands (*14*, *27*), where spectral response of vegetation may be attenuated by flooding and nonvegetated backgrounds (*27*–*29*) and diversity-ecosystem function relationships may vary in space and time (*30*–*32*). At the same time, the unique setting of wetlands as land-water interfaces may support particularly high levels of taxonomic, functional, and phylogenetic diversity (*33*, *34*) and important ecosystem services such as carbon sequestration, hydrological regulation, and coastal protection (*35*–*38*). Unveiling the relationships among biodiversity, phenology, and stability in such systems can support their planning, restoration, and management and inform future studies in the face of wetland and biodiversity loss (*6*, *27*, *32*, *36*).

**Fig. 1.**
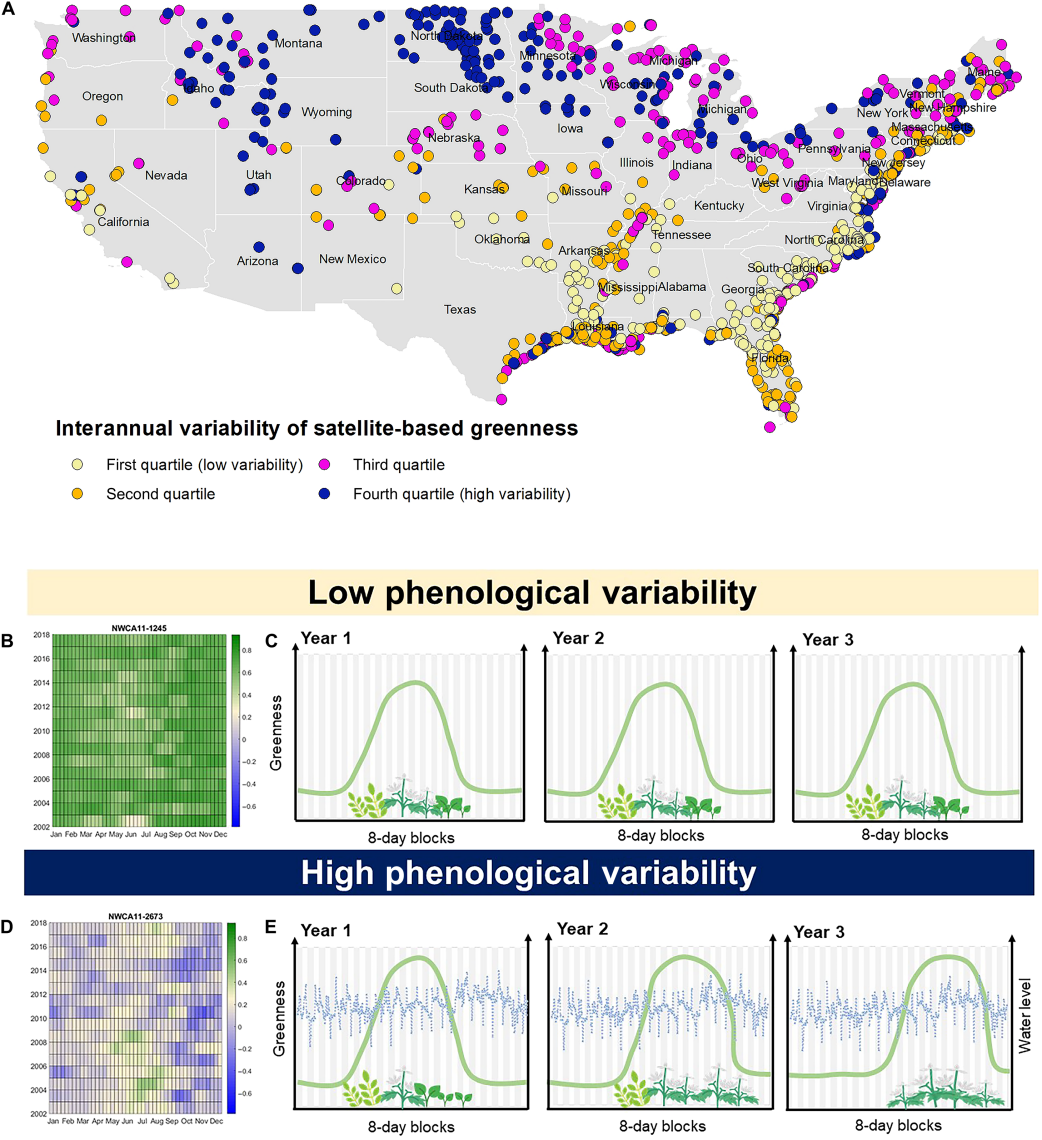
Satellite-based indicator of wetland phenological variability and its geographic variation. Phenological variability is computed from the 2002–2009 time series of NDVI data based on 250-m spatial resolution, 8-day temporal resolution MOD09Q1.006 satellite spectral reflectance product: (**A**) Spatial distribution of phenological variability measure across of 1138 wetlands included in the 2011 NWCA, (**B**) example of 2002–2019 NDVI time series for a site with a low phenological variability (color legends show non–gap-filled NDVI [−1;1] values for the 8-day periods within each year), (**C**) conceptual visualization of phenological variability in remote sensing signals with more stable cycles of greenness in a site with a low phenological variability, (**D**) example of 2002–2019 NDVI time series for a site with a low phenological variability, and (**E**) conceptual visualization of phenological variability in remote sensing signals with less stable cycles of greenness and daily fluctuations in water levels.

In response to this call, here, we develop an indicator of phenological variability and test its relationships with taxonomic vascular plant diversity in 1138 wetlands surveyed by the National Wetland Condition Assessment (NWCA) across the conterminous United States (table S1) (*39*). Phenological variability of greenness, computed from an 18-year series of gap-filled (fig. S1) Moderate Resolution Imaging Spectroradiometer (MODIS) satellite–based normalized difference vegetation index (NDVI) values (Fig. 1), represents cumulative deviation of wetland NDVI values from their long-term means summarized over 46 8-day seasonal steps of each year. We hypothesized that plant diversity is negatively associated with interannual variability in NDVI because more diverse communities may experience more diverse responses to environmental fluctuations, greater asynchronies in species resource use, and longer growing seasons, as shown by previous studies (*2*, *4*, *8*, *12*). We first assessed this hypothesis using multivariate statistical regression models, which tested both plant diversity and a suite of covariates, i.e., variables that may contribute to phenological variability independently of plant diversity (table S2). The latter included indicators of climate and its variability, site-specific environmental conditions, and potential spectral artifacts related to tall vegetation, flooding, and disturbance (table S2 and fig. S2). Then, we compared the relationships between diversity and phenological variability to diversity relationships with other stability measures, specifically, ratios of annual mean to SD of satellite-derived NDVI, net primary productivity (NPP), and gross primary productivity (GPP) (hereafter “stability of annual greenness” measures; table S3). Last, we used structural equation modeling (SEM) to test for direct and indirect effects of plant diversity on phenological variability and stability derived from the same satellite greenness product.

## RESULTS

### Plant diversity negatively correlates with phenological variability in the presence of covariates

**Fig. 2.**
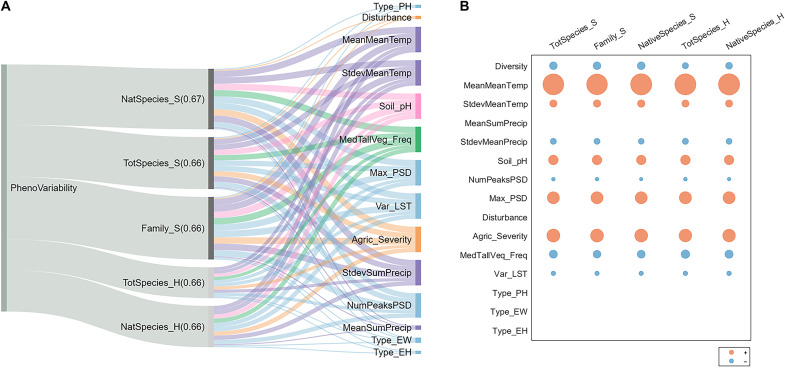
Inclusion of plant diversity metrics and landscape covariates in multivariate regression models predicting phenological variability. Potential predictor variables in regression models include plant richness (total and native: TotSpecies_S and NatSpecies_S, respectively), family richness (Family_S), Shannon-Wiener diversity for all (TotSpecies_H) and native (NatSpecies_H) species, and covariates (table S2), while phenological variability (PhenoVariability) is the dependent variable. (**A**) Sankey diagram representing inclusion of plant diversity and covariates in models within 2 units of the lowest Akaike information criterion (AIC_c_) for each diversity metric; width of the gray ribbons is proportional to the number of such models (maximum = 6 and minimum = 3), while colored ribbons are associated with covariates (table S2) and their widths are proportionate to the frequency of a given covariate’s inclusion in the lowest-AIC_c_ models for each diversity metric, regardless of statistical significance. (**B**) Bubble chart showing standardized regression coefficients for variables included in the lowest-AICc model for each diversity metric where blue coefficients stand for negative effects and pink-colored coefficients stand for positive coefficients. Detailed information on coefficient standard error in the lowest-AIC_c_ models for phenological variability and stability in annual mean and maximum NDVI is provided in table S3. The number in parentheses next to diversity metrics indicates the adjusted coefficient of determination (*R*^2^) of the lowest-AIC_c_ model for that metric. For variables shown in (A) but missing from (B), regression coefficients were not significant at *P* = 0.05 level in the lowest-AIC_c_ models.

All selected plant diversity metrics [total (TotSpesies_S; fig. S3A) and native (NatSpecies_S) species richness, family richness (Family_S), and Shannon-Wiener diversity index for all (TotSpecies_H) and native (NatSpecies_H) species; table S1] were included in the highest-support regression models (Fig. 2, A and B, and table S3) within the two lowest units of sample size–adjusted Akaike information criterion (AIC_c_) (*40*). In these models, plant diversity showed a negative association with phenological variability but only in the presence of several covariates (Fig. 2B and tables S2 and S3). Mean annual atmospheric temperature (MeanMeanTemp; fig. S3B) was positively related to phenological variability in regression models (Fig. 2B); however, its relationship was stronger when using a second-order polynomial transformation (table S3), indicating less stable phenology in both colder and warmer climates (Fig. 1A, fig. S3B, and table S3). Variation in annual precipitation (StdevSumPrecip; fig. S3C), number of significant cycles in greenness series (NumPeaks), frequency of tall vegetation (MedTallVeg_Freq), and variability in land surface temperature (Var_LST) also showed significant negative, although weaker, associations with phenological variability (Fig. 2B and table S3).

In contrast, SD in annual temperature (StdevMeanTemp; fig. S3D), soil pH (Soil_pH), indicator of the cyclicality in greenness (Max_PSD; table S2), and presence of agricultural stressors based on wetland adjacency to agricultural areas (Agric_Severity; table S2) positively correlated with phenological variability (*P* < 0.05 for all; Fig. 2B and table S3). Total annual precipitation (MeanSumPrecip; fig. S3E) was not often selected in the lowest-AIC_c_ models; however, it strongly and positively correlated with its SD [Pearson’s correlation coefficient (*r*) = 0.89, *P* < 0.001; table S5]. Wetland type determined by NWCA hydrological status and vegetation [palustrine woody (Type_PW), palustrine herbaceous (Type_PH), estuarine woody (Type_EW), and estuarine herbaceous (Type_EH) and NWCA-assigned disturbance status (disturbance)] were included less often and not significantly related to phenological variability in the highest-support models (Fig. 2, A and B, and table S3). Residuals of the lowest-AIC_c_ models showed several hot spots, representing more significant than by chance grouping of both high and low values (see example for species richness in fig. S3F). Most of these hot spots were in coastal regions (fig. S3F).

### Diversity and covariates better explain phenological variability than stability of annual greenness

**Fig. 3.**
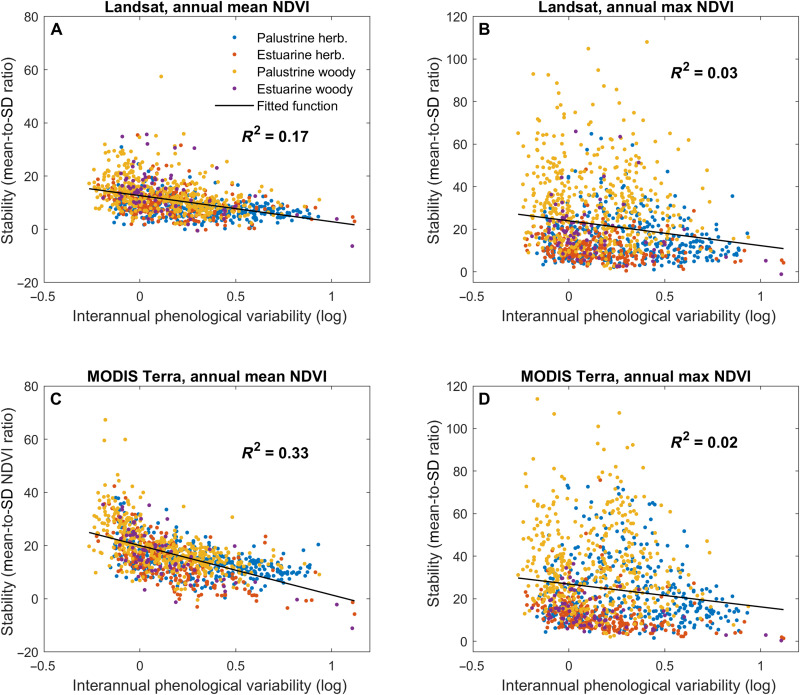
Relationships between phenological variability and alternative measures of stability in annual greenness. Measures of stability represent 2002–2019 mean-to-SD ratios of the NDVI computed as follows: (**A**) annual mean based on the Landsat surface reflectance, (**B**) annual maximum based on the Landsat surface reflectance, (**C**) annual mean based on the MODIS Terra satellite 8-day surface reflectance product MOD09Q1.006 used in computation of phenological variability, and (**D**) annual maximum based on the 8-day MODIS Terra surface reflectance MOD09Q1.006 product. Here, high values of phenological variability on the horizontal axes indicate a high variability in magnitudes of 8-day NDVI products over multiple years, while high stability values indicate a small variation in annual average (A and C) and maximum (B and D) NDVI values over time. Each scatter plot is fitted with a linear function and shows the *R*^2^ value. The legend indicates wetland types determined by NWCA based on hydrology and dominant vegetation (*39*): palustrine herbaceous, estuarine herbaceous, palustrine woody, and estuarine woody.

Phenological variability showed a weak nonlinear negative correlation with stability indicators computed as mean-to-SD ratios for satellite-derived annual greenness and productivity (Fig. 3 and table S4). Diversity and covariates were also important in models for stability of minimum and maximum annual greenness (table S3 and fig. S4), consistent with previous studies (*5*). However, inclusion of covariates somewhat differed from the models for phenological variability (table S3), showing, in particular, a greater importance of wetland types (tables S2 and S3) defined by hydrology (estuarine and palustrine) and dominant vegetation (woody and herbaceous). However, highest-support models for all 14 tested stability measures explained up to 15 to 66% less variation than models for phenological variability (tables S3 and S4), with the weakest fit for stability in GPP and NPP (table S4). The latter result could be affected by the incomplete inclusion of NWCA wetlands in GPP and NPP spatial datasets (table S4), likely because of product-specific land masking missing a number of estuarine wetlands (fig. S5), also contributing to the uneven correlation among them (table S5). Thus, although phenological variability may be more sensitive to the status of biomass manifested in annual NDVI summaries than of annual productivity indicators, this assertion warrants caution because of different sample sizes and uneven representation of wetlands among spatial datasets.

### Diversity mediates stability of greenness via phenological variation

**Fig. 4.**
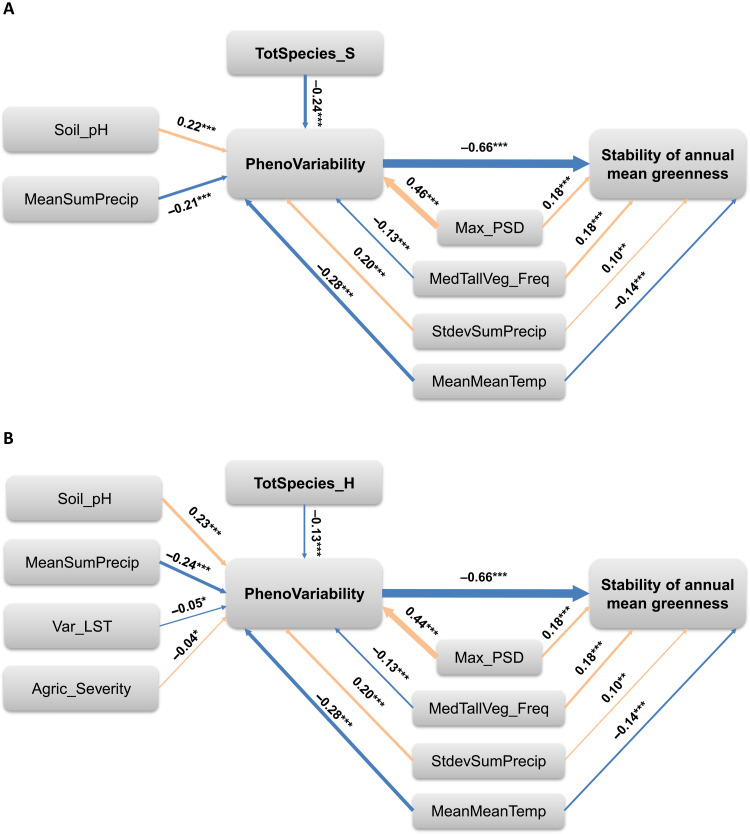
Path analysis diagram representing effects of plant diversity and covariates on phenological variability and the stability of mean greenness. (**A**) The effects of total plant species richness (TotSpecies_S) and (**B**) the effects of Shannon-Wiener diversity of all species (TotSpecies_H) on phenological variability (PhenoVariability) and indirect effects on stability of annual mean greenness, accounting for mean atmospheric temperature (MeanMeanTemp), SD in annual total precipitation (StdevSumPrecip), frequency of tall vegetation (MedTallVeg_Freq), soil pH (Soil_pH), variation in LST (Var_LST), agricultural stressors (Agric_Severity), and cyclicality in remote sensing signals of wetland sites (Max_PSD) (definitions are in table S2). Width of connecting arrows is proportional to standardized path coefficients (regression weights *r*) shown as numerical labels. Other variables and path relationships were tested but removed if their path coefficients were not significant. *P < 0.05, **P < 0.01, and ***P < 0.001.

Path analysis with SEMs found that in the highest-support models, plant species richness (TotSpecies_S) was negatively related to phenological variability (*r* = −0.24, *P* < 0.001; Fig. 4A and table S6), while the latter, in turn, had a strong negative association with stability of greenness computed for annual mean NDVI (*r* = −0.66, *P* < 0.001; Fig. 4A). In contrast, species richness and stability of annual greenness were not significantly related (*P* > 0.05). Shannon-Wiener diversity (TotSpecies_H) showed a negative and significant but weaker association with phenological variability (*r* = −0.13, *P* < 0.001; Fig. 4B), and its direct relationship with stability of greenness was also not significant (*P* > 0.1). Similar effects were observed also in the highest-support SEMs for family richness and for Shannon-Wiener diversity of native species (table S6). However, in models with native species richness, diversity also had a significant, although weak, direct negative relationship with phenological variability (*r* = −0.072, *P* = 0.022).

Alternative models testing whether phenological variability, stability of greenness, and/or covariates affected plant diversity were not supported, showing significant difference between the model-implied and observed structure of the data with the probability of corresponding chi-square statistics substantially below 0.05 and the ratio of chi-square to degrees of freedom exceeding 3. In addition, despite a comparable fit between multivariate regression models for stability of annual maximum NDVI and those for annual mean NDVI (tables S3 and S4), SEMs using stability of annual maximum NDVI to test similar hypotheses were not sufficiently supported (showing the ratio of chi-square to degrees of freedom greater than 3). Together, these outcomes suggest that interannual phenological variability may be a mediator in plant diversity relationships with stability of biomass when the latter is indicated by the annually averaged greenness.

Significant paths in SEMs (Fig. 4, A and B, and table S6) involved several covariates that consistently showed strong associations with phenological variability and, in some cases, stability of annual greenness (table S6). In the SEM with species richness (Fig. 4A), both mean atmospheric temperature and mean annual precipitation had a negative effect on phenological variability (*r* = −0.28 and −0.21, respectively, *P* < 0.001 for each; Fig. 4A), while variation in annual precipitation had a strong positive effect (*r* = 0.20, *P* < 0.001). However, the associations of mean atmospheric temperature and mean annual precipitation with stability of greenness were also negative and significant but weaker than those for phenological variability (*r* = −0.09 and −0.11 and *P* = 0.009 and 0.005, respectively; Fig. 4, A and B). Together, these results imply that, in warmer, wetter climates, NWCA wetlands were likely to show more stable phenology of greenness but not necessarily more stable annual biomass.

Among other factors, soil pH had a strong positive effect on phenological variability (*r* = 0.20, *P* < 0.001), suggesting lower variation in greenness phenology in more acidic wetlands. Last, Max_PSD, related to magnitude of the main cycles in greenness fluctuations, showed a very strong positive effect on phenological variability (*r* = 0.46, *P* < 0.001) and a somewhat weaker but significant positive effect on stability of greenness (*r* = 0.15, *P* < 0.001). This evidence suggests that wetlands with greater magnitude of greenness fluctuations may exhibit both less stable seasonality and less stable interannual biomass.

Contrary to expectations, variability in LST was not significantly related to either phenological variability or stability of greenness in SEMs using total or native species richness and family richness (*P* > 0.05 for all; Fig. 4A and table S6). However, it showed a weak but significant positive association with phenological variability in SEMs using total and native Shannon-Wiener diversity (*r* = −0.05, *P* = 0.032; Fig. 4B and table S6), consistent with the expectation that more variable surface thermal conditions could contribute to interannual variation in vegetation seasonality. SEMs using Shannon-Wiener diversity also included a significant path indicating positive relationship between agricultural stressors and phenological variability (*r* = −0.04, *P* = 0.044; Fig. 4B).

Overall, across SEMs using different diversity metrics, standardized path coefficient for plant diversity and phenological variability relationship were comparable to those of most other variables including climatic and soil properties (Fig. 4, A and B, and table S6), indicating that phenological variability was simultaneously influenced by multiple factors and that plant diversity was an important driver among those.

## DISCUSSION

### Biodiversity effect on stability of biomass production may be mediated by phenological dynamics

Our results indicate that wetland plant diversity is linked with temporal stability of both biomass production and phenological variation at broad geographic scales, consistent with earlier studies in drylands (*5*) and other terrestrial ecosystems (*12*). Significant negative effect of plant diversity on phenological variability in the presence of key covariates concurs with the earlier discussed stabilizing effects of diversity on ecosystem function via enhanced redundancy and diversity of responses to disturbance and stressors (*2*, *5*, *12*). These effects imply that wetlands with more diverse vegetation may be more likely to develop stabilizing mechanisms including asynchronies and different adaptations to various environmental conditions within the growing season, which allows species to take advantage of “temporal” ecological niches as discussed in other systems (*23*, *24*). Stabilizing mechanisms could also result from the unique aspects of wetland hydrological connectivity and species dispersal within their landscape networks (*41*), enabling particular adaptive opportunities such as genetically diverse seed banks promoting rapid responses in regions with high environmental variability or after pulse disturbance. Higher standardized regression weights for species richness (Fig. 4A) than those of Shannon-Wiener diversity (Fig. 4B) suggests that asynchrony within NWCA wetland communities in our sample may be more important than variation in species-level population abundance (*21*, *42*). This assertion should be tested in the future with multitemporal vegetation observations.

Our findings further show that contributions of wetland plant diversity to satellite-derived stability indicators could be mediated by phenological consistency, suggesting that some of the stabilizing pathways of plant diversity could lie in its effects on seasonality of ecosystem function (Fig. 4, A and B, and table S6). This evidence is consistent with the findings from the terrestrial ecosystems of Switzerland (*12*), where biodiversity showed a strong indirect effect on productivity via lengthening of the growing season and a significant but weaker direct effect. It was proposed that these effects may result not only from temporal complementarity of species promoting their coexistence (*43*) but also from the potential of biodiversity to promote the use of additional niche space created by changes in climate or other environmental factors (*12*), making biodiversity a key player in phenological sensitivity to environmental change (*44*). In the context of our study, the latter assertion should be examined in more detail with repeated wetland surveys, such as future NWCA data releases.

Given common zonation of vegetation species and traits along wetland inundation gradients (*33*, *45*), mechanisms behind the links between diversity and phenological variability could include seasonal asynchrony and diversity of responses to flooding, climatic variation, and disturbance (*4*), facilitating complementary utilization of space and resources. However, this assertion requires further research, as earlier studies do not reach a consensus on diversity-complementary relationships, finding them relevant mainly at lower species richness levels (*46*–*48*). Our results also contrast with the evidence from remote sensing–based study of wetland phenology in Canada (*14*), where plant assemblage diversity together with climatic and edaphic factors played a stronger role in growing season length and variability than species richness of individual communities. Advancing this knowledge thus requires expanding beyond the taxonomic diversity to investigate the role of functional traits and phylogenies in phenological patterns and functional stability (*4*, *8*, *22*).

### Both phenological drivers and spectral artifacts are relevant to plant diversity-stability research in real-world landscapes

Similar to earlier research (*4*, *5*, *8*, *12*), our results show that in real world, non-experimental landscape phenological variability responds to a number of environmental drivers and data artifacts in addition to plant diversity (*4*, *14*, *27*–*29*). In particular, the importance of climate factors in regression models and SEMs is consistent with the effects of climate on timing of biomass production and development (*5*, *8*) and opportunities for temporal niche utilization within the growing season that may vary year to year (*12*, *14*). Negative effects of both temperature and precipitation and positive effect of variation in precipitation on phenological variability in SEMs (Fig. 4, A and B) may reflect the potential of climate regimes to regulate growing season phenology (*12*, *14*) and hydrological processes (*41*) and provide more temporal niche opportunities in warmer, wetter climates.

Environmental factors may also shape vegetation adaptations that affect timing, magnitude, and spectral characteristics of their phenological changes (*5*, *12*, *14*). For example, the negative effect of soil pH on phenological variability (Fig. 2B and table S3) likely captures environmental constraints on plant diversity and adaptations related to geographic setting. Lower phenological variability at more acidic, i.e., low-pH, sites may reflect adaptations to nutrient limitations, including perennial habit and evergreenness (*32*) and, possibly, the constraints on the overall plant biomass and coverage, translating into lower interannual variation in greenness holding other factors constant. Similarly, the importance of medium-tall vegetation frequency in the higher-support models with a negative effect on phenological variability (Fig. 2, A and B, and table S3) may reflect spatial dominance (*30*, *31*) of taller woody plant canopies, affecting greenness and its year-to-year similarity by obstructing understory vegetation and background flooding.

Unlike field observations of phenology at the level of individual species, remotely sensed phenological patterns represent signals from multiple cover types integrated within image pixels. Wetland hydrological dynamics may influence both the seasonality of wetland plant biomass and the ability of satellite products to adequately represent the latter (*26*, *28*). Consistent selection of both maximum power spectral density (PSD) of the greenness time series and variation in LST in the highest-support regression models (Fig. 2, A and B, and table S3) and SEMs (Fig. 4, A and B) indicates the importance of such covariates, which may be simultaneously sensitive to both vegetation processes and spectral artifacts from flooding and background reflectance (*27*, *28*). Positive association between maximum PSD and phenological variability (Figs. 2B and 4, A and B) implies that, from a remote sensing perspective, wetlands are less phenologically stable when their main greenness cycle involves a higher magnitude of seasonal change, e.g., in predominantly deciduous ecosystems. Low maximum PSD in dynamic tidal systems, particularly herbaceous, reflects the amplification of their spectral variability by short-term flooding, thus signaling spectral effects of inundation in the absence of site-specific hydrological data. Negative statistical effect of variability in LST on phenological variation in regression models (Fig. 2B and table S3) and SEMs (Fig. 4B and table S6) could result from more pronounced thermal variability in regions experiencing snow and ice coverage with variable seasonal timing of snowmelt. Significant effect of variability in LST on phenological variability in SEMs with Shannon-Wiener diversity (Fig. 4B and table S6) in contrast to those with species and family richness (Fig. 4A and table S6) further suggests the importance of vegetation abundance in phenological sensitivity of satellite-derived thermal and greenness indicators.

In relation, coarser spatial resolutions of satellite products increase the sensitivity of pixel-level greenness to non-wetland backgrounds and their seasonality. Agricultural systems may exhibit substantial interannual differences in crop planting and growth schedules, which may amplify long-term variation in the seasonality of greenness. The importance of the agricultural stressor severity (*39*) covariate (Fig. 2, A to C, table S2, and fig. S3) was consistent with this expectation, suggesting that the 250-m satellite pixel size can be prone to such effects in wetlands surrounded by agriculture.

### Implications, future research needs, and prospects for biodiversity indicators

Our study provides new evidence on the links between plant diversity and phenological variability in wetland ecosystems at the national scale. Stronger association of plant diversity with wetland phenological variability than with stability of biomass indicators points to potential interplays between diversity and timing of ecosystem function that are not captured by the traditional satellite-based indicators of stability. Furthermore, our results suggest that phenological variability may be a pathway by which plant diversity contributes to stability of ecosystem functions such as biomass production and therefore should be investigated more in depth in wetlands and other ecosystems.

These findings are of high relevance for future research for several reasons. First, they raise a pertinent question whether remotely sensed stability of phenological timing provides a stronger indicator of shifts in biodiversity and ecosystem function than traditional mean-to-SD ratios in annual summaries of greenness. Although our path analysis with SEMs did not corroborate direct effects of phenological variability on diversity (table S6), phenology has long been discussed as one of the essential biodiversity variables that could be monitored with the help of remote sensing data (*19*, *49*) and could indicate both seasonal asynchronies and longer-term biomass fluctuations in plant communities. Exploring this potential requires systematic repeated surveys of plant diversity, as well as more research on which covariates of diversity should be controlled for the given disparities among the scales of field surveys and satellite observations.

Second, an important question arises whether disruptions of stability triggered by biodiversity loss (*6*, *9*) may be manifested in phenological mechanisms, particularly loss of phenological complementarity or amplification of asynchronies with cascading effects across taxa and trophic levels (*17*). From this perspective, biodiversity-phenology relationships, understudied in real-world landscapes (*22*), could help reduce uncertainties in models of ecosystem function that still fall short in capturing seasonality (*50*). These questions, again, argue for more rigorous analyses comparing remotely sensed phenology with field measurements of functional and phylogenetic diversity, which have been particularly scarce in wetlands (*32*, *45*).

Last, our results highlight several sources of uncertainty that need to be considered and addressed by future investigations, bridging remote sensing, geospatial data, and field ecological observations. The presence of hot spots of significant spatial association in regression model residuals (fig. S3F) suggests that future efforts should consider other covariates of phenological variability not captured by our data. Of particular importance can be variables representing hydrological connectivity among wetland sites, which may influence synchronies of their ecological processes (*41*). Limitations of the single-time wetland survey data suggest the need to compare phenological variability with changes in field-measured plant diversity to better understand their coupled dynamics and control for less time-variant site and geographic factors. Last, more research is needed to understand which spatial scales in real-world landscapes are most relevant to diversity effects on phenology, including optimal scales of remote sensing data (*4*, *8*, *22*). This question is especially critical in light of the limited spatial resolution of satellite products in our study, which is prohibitive for understanding local spatial heterogeneity of vegetation within wetland sites. Recent improvements in spatial, temporal, and spectral richness of remote sensing information (*22*) can help more strategically align field- and satellite-based vegetation analyses to address these uncertainties and continue advancing mechanistic understanding of the links among biodiversity, phenology, and ecosystem function.

## MATERIALS AND METHODS

### Wetland sites and plant diversity

This study focused on 1138 conterminous U.S. wetland sites surveyed in the 2011 NWCA by the U.S. Environmental Protection Agency, where the sampling design aimed to represent the broader population of the nation’s wetlands (*39*, *41*). Each site was surveyed at least once during the spring-summer 2011 to record species composition and vegetation structure by form and environmental conditions; for sites with two seasonal visits in 2011 (<10% observations), we used the first survey (*27*, *32*). At each site, sampling was performed in five 100-m^2^ plots within a ~0.5-ha (40 m radius) assessment area (*27*, *32*, *39*), which, in turn, was nested in larger representative area with a radius of ~140 m (*39*). Five indicators of vascular plant taxonomic diversity computed at the site level were used as the main statistical predictors of phenological variability: total (TotSpecies_S) and native (NatSpecies_S) species richness (log transformed), total family richness (Family_S; log transformed), and total (TotSpecies_H) and native species (NatSpecies_H) Shannon-Wiener diversity index (table S1).

### Phenological variability

Wetland greenness was approximated using the popular NDVI ([−1;1]) (*5*, *27*, *32*), computed as the normalized difference of surface reflectance between red (620 to 670 nm) and near-infrared (841 to 876 nm) electromagnetic regions. Surface reflectance data were obtained from the MODIS product MOD09Q1.006 Terra Surface Reflectance at 250-m spatial resolution, summarized for 8-day blocks with 46 site-level data values per year (the timing of which was assumed to be nearly equivalent among different years)

$$\text{NDVI}=\frac{\text{sur}\_\text{refl}\_b02-\text{sur}\_\text{refl}\_b01}{\text{sur}\_\text{refl}\_b02+\text{sur}\_\text{refl}\_b01}$$ (1)

where sur_refl_b01 and sur_refl_b02 represent spectral reflectance in red and near-infrared electromagnetic regions, respectively. Before computing NDVI, we applied cloud masking to the MODIS images using a quality band value of 0 to preserve only “clear” pixels. We further summarized NDVI as site averages of pixels within each wetland site by using Google Earth Engine cloud-based computing interface (*51*) and applied a smoothing approach using Whittaker filtering (*52*, *53*) to fill data gaps and reduce noise (for details, see fig. S1 and caption text). Then, we computed interannual phenological variability of greenness for each wetland site as the cumulative deviation in site-level NDVI from the mean value of its respective 8-day block during 2002–2019

$$\text{Phenological}\;{\text{variability}}_{\text{site}}=\sqrt{\sum_{i=1}^{46}{\left(\frac{\sum_{j=2002}^{2019}({\text{Greenness}}_{i,j}-{\bar{\text{Greenness}}}_{i})}{{\bar{\text{Greenness}}}_{i}}\right)}^{2}}$$ (2)

where Greenness*_i,j_* is the NDVI value for a given seasonal 8-day block *i* and year *j* in the 2002–2019 series if NDVI ≥ 0 and 0 is NDVI < 0. Here, we suppressed NDVI values <0 by forcing them to be equal to zero to reduce the effect of spectral variation in nonvegetated wetland surfaces (with low NDVI by design of this index) on phenological variability estimation and more reliably characterize the latter in terms of vegetation contributions. Note that in (*2*), we also normalized the differences of annual NDVI values within each time block *i* from their mean by also dividing them by their block’s mean value over the 18-year series to enable comparison between sites with a naturally high greenness (e.g., evergreen) or a low baseline (e.g., permanent flooding) and sites where equivalent 8-day time blocks may differ in their potential magnitudes of greenness change (e.g., deciduous versus evergreen). For the subsequent regression analyses, phenological variability estimated using (*2*) was further logarithmically transformed to address the skewness of its distribution. We assumed that higher values of phenological variability represented lower interannual consistency of greenness transition timing, while lower phenological variability indicated greater interannual consistency.

### Alternative measures of stability in greenness

To assess the relevance of phenological consistency to the stability of ecosystem function, we compared our metric of phenological variability (*2*) with the long-term mean-to-SD ratios of 14 satellite-derived proxies of ecosystem function (table S4), representing annual ecosystem GPP and NPP and annual average and maximum greenness as an indicator of aboveground biomass (*5*) and productivity (*12*). While these alternative metrics do not capture the stability of phenological timing, their positive associations with plant diversity measures had been previously discussed as the evidence of stabilizing effects of diversity on ecosystem function [e.g., (*5*, *12*)]. In this analysis, we assessed several forms of such satellite-based measures representing different satellite-based products (i.e., MODIS and Landsat), spatial resolutions, and indicators of ecosystem function (GPP and NPP and biomass), as well as somewhat different spatial coverage with some products not covering the full scope of NWCA wetlands (table S4).

### Regression analyses

We assessed the association between phenological variability and plant diversity of NWCA wetland sites using generalized linear regression modeling with phenological variability (PhenoVariability) as a response variable, plant diversity as the main predictor variable, and several covariates (i.e., variables expected to influence phenological variability independently of diversity) described below. The main goal of this analysis was to examine the importance of diversity at the broad scale of NWCA data after adjusting for the relevant covariates identified through the most parsimonious models as discussed below. We tested regression models with different combinations of plant diversity, and covariates (*N* = 32,767) were conducted in MATLAB software v.2022a (MathWorks Inc.) using the function fitlm. The models were subsequently ranked using a form of the AIC_c_ (*40*). The same models were also assessed for each of the alternative measures of stability (table S4). Residuals of the lowest-AIC_c_ models were tested for spatial association using Getis–Ord Gi statistics (*54*) and the corresponding Hot Spot Analysis tool in ArcGIS Desktop v.10.8 (Esri Inc.). Last, to help interpret the statistical effects of diversity in the presence of covariates, we assessed how often the latter were selected in the models with the highest statistical support (i.e., within 2 units of AIC_c_ from the model with the lowest AIC_c_). We then conducted similar regression model comparisons to test the associations of plant diversity and covariates with each of the 14 alternative measures of stability (table S3).

### Covariates

Several covariates were included in regression modeling to account for environmental factors, site conditions, and remote sensing artifacts contributing to phenological variability in addition to plant diversity (table S2). These included proxies of climate, local soil properties, cyclical variability in the greenness time series, wetland hydrological type, prevalence of medium-tall vegetation, NWCA-determined disturbance status, and severity of agricultural stressors in the wetland buffer area (table S2).

Climate covariates were included to adjust for climate-induced variability in phenological timing of changes in greenness (*12*, *15*, *18*, *45*, *55*) using gridded datasets (table S2) for daily mean atmospheric temperature and daily precipitation over 2002–2019, accessed via Google Earth Engine. For each site, atmospheric temperature was summarized for 2002–2019 as mean and SD of its annual mean value (MeanMeanTemp and StdevMeanTemp, respectively), while precipitation was summarized as 2002–2019 mean and SD of its annual sum (MeanSumPrecip and StdevSumPrecip, respectively).

Soil pH from NWCA field surveys (*39*) was included to account for potential effects of site conditions on soil quality, nutrient availability, and, consequently, physiological characteristics and adaptations of vegetation that could influence the magnitude and stability of greenness in addition to diversity per se (*27*, *32*). This variable was also expected to represent variability in soil properties among different wetland types located in similar climatic regions.

### Proxies of variability in greenness due to vegetation, hydrology, and background effects

Hydrological attenuation and spectral reflectance from nonvegetated background surfaces may reduce the degree to which greenness values represent vegetation at a given pixel scale (*27*, *28*). Local hydrological factors may also contribute to local microclimatic conditions in wetlands and thus, potentially, the timing of plant phenology. However, accounting for these effects directly requires frequent, site-specific observations of both hydrology and vegetation biomass, which are not currently monitored at a national scale. To circumvent this challenge, we tested several relevant indicators as model covariates. Three of them were binary variables capturing four major NWCA wetland types based on hydrology and vegetation, with palustrine herbaceous, estuarine woody, and estuarine herbaceous included in the models (table S2), with palustrine woody treated as a reference. We expected that phenological variability would be greater in estuarine wetlands compared to palustrine ones because of the influence of tidal inundation on spectral signals and that these effects would be more pronounced in herbaceous marshes where inundation is more visible to remote sensors than in woody sites.

We included another indicator that represented variability in LST (Var_LST) computed from MOD11A2v.6 LST product at 1-km spatial resolution. To address the key challenge of temporal gaps in 1-km LST due to atmospheric effects, we developed a specialized gap filling approach. For each wetland site, we first identified missing values and assessed whether they formed consecutive sequences and of which length. Given the uncertainty associated with filling very large gaps, we applied gap filling only to sequences of no more than four consecutive missing values; sequences of five and more consecutive missing values were left as missing data. For portions of wetland time series that contained missing values and qualified for gap filling based on these criteria, we used MATLAB function fillmissing with the space-preserving piecewise cubic spline interpolation (“pchip” fill method of this function).

Next, we computed variability in LST as the average deviation in LST of the satellite pixel overlapping a wetland site from the mean value of its respective 8-day block during 2002–2019, corrected for the number of 8-day blocks that could not be robustly gap filled

$$\text{Variability}\_{\text{LST}}_{\text{site}}=\sqrt{\frac{\sum_{i=1}^{46}{\left(\frac{\sum_{j=2002}^{2019}({\text{LST}}_{i,j}-{\bar{\text{LST}}}_{i})}{{\bar{\text{LST}}}_{i}}\right)}^{2}}{N_{\text{valid}}}}$$ (3)

where LST*_i,j_* is the LST value for a given seasonal 8-day block *i* and year *j* in the time series and *N*_valid_ is the count of seasonal 8-day blocks containing valid data (i.e., not consisting entirely of missing values).

We expected that greater variability in LST would be associated with greater phenological variability of greenness due to the variation in microclimate-related triggers of phenological events such as snowmelt (*56*). We also expected that, given the potential contrasts in LST between land and water surfaces, including its variability as a covariate could help partially account for fluctuations in greenness due to variability in flooding and its effects on wetland spectral signals.

We also included two covariates characterizing cyclical variability in greenness time series (table S2 and fig. S2). Such cyclical variability results from seasonal changes in vegetation biomass and flooding attenuation, which varies with plant height and cover (*28*), as well as potential effects of recurring stressors, disturbance, or management. Cyclical variability of greenness was characterized using periodogram analysis of the full 2002–2019 NDVI time series of each wetland site (fig. S1). Periodogram analysis estimates the spectral density of a signal in the time series, which reveals how the signal strength of different cycles (represented by a power spectrum measure) varies with temporal frequencies of these cycles (*57*, *58*). Hence, this approach identifies temporal frequencies associated with the most pronounced periodic events in the time series (*59*) characterized by significantly higher power spectrum density magnitudes than other variation assumed to be signal noise (fig. S2).

Such variability effectively means that NDVI time series are likely to differ among individual wetlands both in the degree of signal noise and in signal frequencies associated with most pronounced changes in greenness (e.g., short-term fluctuations prevalent in herbaceous tidal marshes with frequent hydrological attenuation versus annual changes in canopy greenness characteristic of nontidal forested wetlands with signals dominated by tree cover).

To perform periodogram analysis, the NDVI time series of each site were first differenced (using MATLAB diff function) to remove potential nonstationarity before periodogram estimation. Next, for each site, we computed periodogram as PSD associated with different frequencies of the signal using Welch’s method (MATLAB pwelch function) (*58*), which provides an enhanced reduction of noise in the series compared to other approaches, although at the expense of the reduced frequency resolution. This approach involves breaking down the time series signal into successive time windows, assessing the periodogram for each window, and then averaging them. We used a 5-year window length to optimize the detection of cycles across a broad range of frequencies while also suppressing the short-term noise.

Potentially high variability in NDVI time series among individual wetlands in geographically broad NWCA sample makes it difficult to establish a single null hypothesis for detecting the most pronounced periodic cycle series from PSD-frequency patterns. For this reason, we identified significant peaks in the resulting PSD estimate as instances where the lower bound of the PSD’s 95% confidence interval exceeded the global mean of the estimated PSD values across all frequencies as a simple yet more generalizable null hypothesis (fig. S2) (*59*). We further tested for presence of clusters of redundant peaks, i.e., groups of significant peaks corresponding to consecutive values of frequencies, separated by frequency ranges with no significant peaks. Because of similar frequency values within such clusters, we used only the maximum peak PSD value as representative cycle associated with that cluster (fig. S2).

On the basis of the periodogram analysis, we estimated two characteristics of cyclical variability in greenness: (i) maximum PSD across all possible signal frequencies (Max_PSD), related to the magnitude of NDVI change within the dominant greenness cycle of a wetland, and (ii) total number of clusters with significant PSD representing the total number of the most pronounced periodic cycles in the time series (NumPeaksPSD). We expected that wetlands with greater Max_PSD would show greater variation in phenological timing year to year due to a greater amount of greenness change associated with such a cycle. We further expected that temporary flooded conditions may affect Max_PSD by increasing the difference in NDVI between high-biomass stages with little attenuation and flooded conditions with low NDVI representative of water. Relatedly, frequent flooding could reduce the amplitude of greenness fluctuations by reducing the probability of remote observations of water-free plant biomass. We further expected that sites showing both lower Max_PSD and greater NumPeaksPSD would exhibit greater overall phenological variability due to more complex time series, such as in hydrologically variable sites.

We also included the combined frequency of medium and tall vegetation summarized at the NWCA site level (MedTallVeg_Freq; table S2). This covariate was used to account for a potentially greater contribution of tall species (particularly woody plants) to site greenness, given a somewhat coarse 250-m spatial resolution of MODIS input data and the potential to obstruct the reflectance from herbaceous vegetation in their understory (*27*, *32*). We therefore expected that greater prevalence of taller plants would have a negative effect on phenological variability due to greater interannual consistency in their dominant contribution to site greenness.

Last, we included two covariates related to disturbance factors (table S2), both of which were expected to increase phenological variability but for somewhat different reasons (*27*, *32*). The first one represented NWCA’s integrated index of disturbance and stress (disturbance) (*39*), converted from the original three-level scale (least, intermediately, and most disturbed) to a binary variable (0, least disturbed; 1, intermediately or most disturbed). The second more specific disturbance covariate represented severity of agricultural stressors near wetland’s assessment area (Agric_Severity), also converted to a binary variable [0, no stressor; 1, stressor(s) of any severity present]. The presence of agriculture-related stressors was expected to increase phenological variability of wetlands as a background effect at MODIS data scale due to potential interannual variation in crop planting schedules and crop-specific seasonality of biomass development. Evidence of such potential agricultural stressors, represented by the adjacency of wetland sites to agricultural areas (table S2), was recorded by NWCA surveys (*39*).

### Path analysis for diversity effects on phenological variability and stability of greenness using SEM

Last, we tested the hypotheses about potential causal relationships among plant diversity, phenological variability, stability of annual greenness, and environmental factors using path analysis with SEMs (*60*). We tested for direct effects of plant diversity on phenological variability and stability of annual greenness, indirect effects where one of these stability measures mediated diversity effect on another, and reverse relationships where plant diversity was influenced by one or both of these stability measures. We constructed SEMs capturing these hypotheses and main potential contributing factors, including climate, soil pH, variation in LST, cyclical variability of greenness, and agricultural stressors, and fitted them using maximum likelihood estimation in IBM SPSS Amos software version 2.7. Similar to (*12*), we started with saturated models and systematically eliminated nonsignificant (*P* > 0.05) paths to select the models where all path coefficients were significantly different from 0. We computed the standardized regression weight coefficients (*r*) to compare model effects and evaluated the fit of the models to hypotheses using (i) the chi-square test (where not significant chi-square values indicate no statistical difference between the hypothesized model and data structure), (ii) the ratio of chi-square to degrees of freedom expected to be <3, and (iii) criteria of fit such as comparative fit index expected to be >0.90 for a reasonably good fit. Models not meeting these criteria were not analyzed further (*60*).

Last, to help interpret the statistical effects of diversity in the presence of covariates, we also compared the regression coefficients of diversity metrics between models with the highest statistical support (within 2 units of AIC_c_ from the model with the lowest AIC_c_) and models in which the diversity variable’s regression β coefficient was not significantly different from the most negative coefficient value, indicating a higher-magnitude stabilizing effect of diversity based on the coefficient 95% confidence intervals.

## Supplementary Materials

### This PDF file includes:

Figs. S1 to S5 Tables S1 to S6 Data S1 to S3 References
