# Supplementary material for: Plant diversity reduces satellite-observed phenological variability in wetlands at a national scale
Source: Sci Adv. 2022 Jul 22;8(29):eabl8214. doi: 10.1126/sciadv.abl8214 (PMC9307251; doi:10.1126/sciadv.abl8214)
Supplement: Supplementary file 1 — Figs. S1 to S5 Tables S1 to S6 Data S1 to S3 References [file sciadv.abl8214_sm.pdf]

Science Advances
AAAS

Supplementary Materials for

Plant diversity reduces satellite-observed phenological variability in wetlands at a national scale

Iryna Dronova *et al.*

Corresponding author: Iryna Dronova, idronova@berkeley.edu

Sci. Adv. **8**, eabl8214 (2022)
DOI: 10.1126/sciadv.abl8214

This PDF file includes:

Figs. S1 to S5
Tables S1 to S6
Data S1 to S3
References

Supplementary Figures

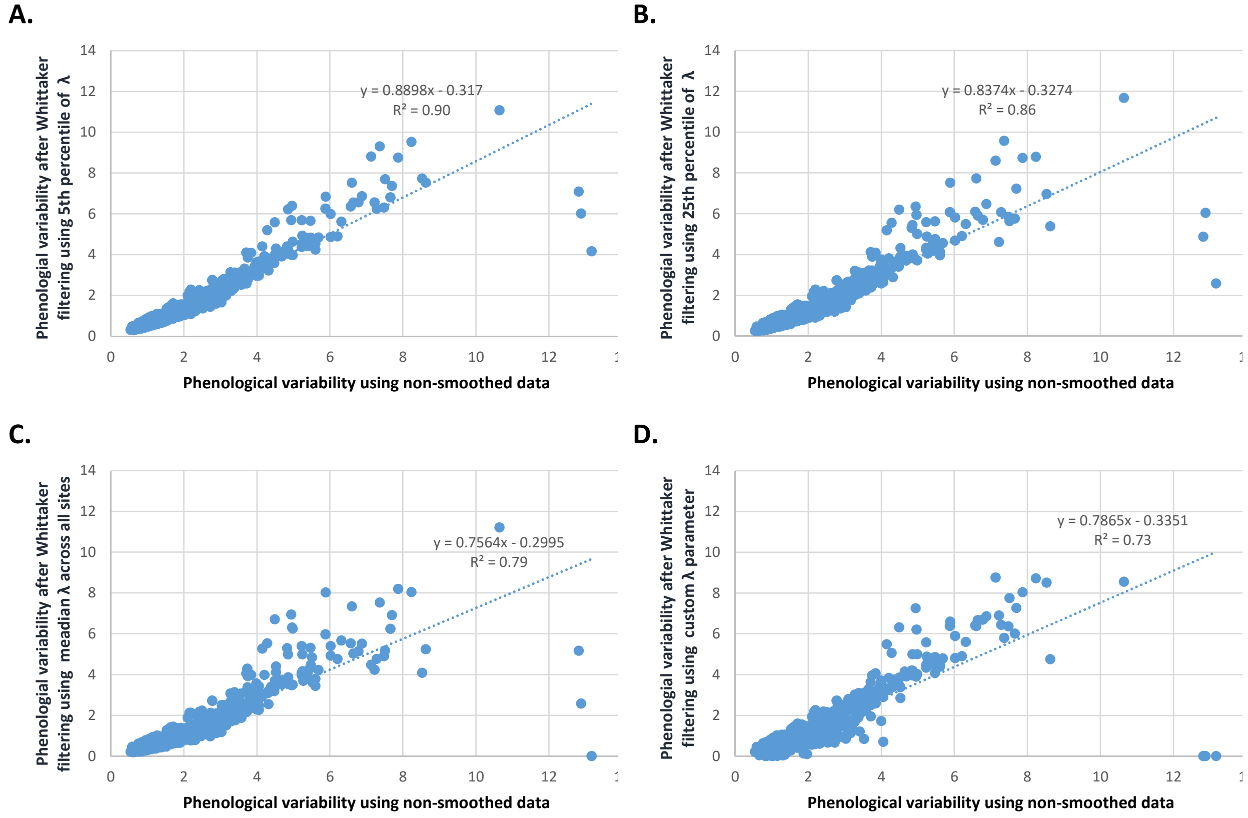

Fig. S1. Examples of correlation between phenological variability computed from non-smoothed 2002-2019 NDVI series and after smoothing using Whittaker filtering with different selections of the smoothing parameter lambda (λ) which controls the strength of smoothing: (A)-(C) using the 5th, 10th, and 25th percentile of lambda values from all wetland sites, respectively, from the optimized values computed for all NWCA wetland time series, and (D) using lambda computed individually for each wetland time series. This assessment incorporated codes for smoothing from the Whittaker Smoother Toolbox developed by Paul H.C. Eilers in 2003 (*52*). **Rationale:** Our subsequent analysis used lambda equal to 5th percentile of all wetland sites to balance the gap-filling objective with the need to avoid extreme smoothing of noisier time series to preserve their natural signal variability. While a number of strategies to objectively select the smoothing parameter have been proposed (*52*), an important consideration from the ecological perspective of our study is preserving the "meaningful" variation representative of changes in ecosystem and vegetation states (*29*, *61*). In the large, heterogeneous NWCA sample, wetlands are likely to differ in their characteristic amount of variability in NDVI time series, which our statistical design already accounts for noise by including covariates sensitive to fluctuations in greenness (Table S2). Using objective estimation to selecting lambda individually for different time series based on common methods (*52*, *62*) thus creates a challenge of the uneven degree of noise removal among wetland sites with different levels of such noise. This figure illustrates that phenological variability estimated from smoothed and non-smoothed series maintained a strong positive correlation both when a single value of lambda was applied to all sites (panels A-C) and when the parameter was optimized individually for wetland sites (panel D). However, these choices affected the magnitude of extreme cases, where three estuarine wetlands with the highest phenological variability based on the non-smoothed values had exhibited substantially lower values after smoothing, particularly if using their individual lambdas. Based on these considerations and statistical design of the study explicitly testing covariates of phenological variability sensitive to noise, we prioritized the gap-filling objective over noise removal and opted for a less extreme lambda value.

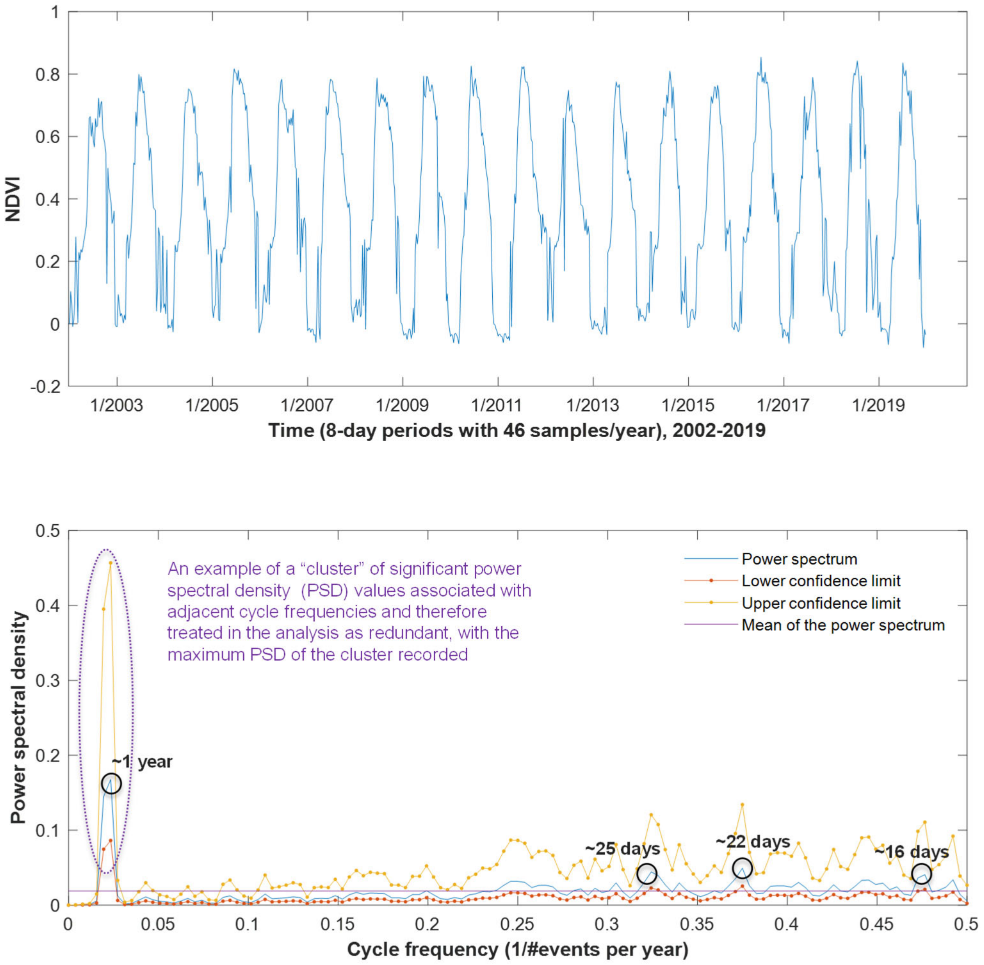

Fig. S2. Illustration of the periodogram analysis used to quantify Max_PSD and NumPeaksPSD covariates. The upper panel shows the original 2002-2019 time series of the Normalized Difference Vegetation Index (NDVI) computed from the 8-day 250m MODIS spectral reflectance product[12] for a sample wetland site. The bottom panel shows the power spectral density (PSD) of the NDVI time series on the vertical axis and its 95% confidence interval limits plotted against the cycle frequency on the horizontal axis. In this Figure's example, the highest PSD value (leftmost peak in the figure) corresponds to the frequency matching the annual cycle of NDVI (with 46 8-day data values from the MODIS products, translating into cycle frequency of ~1/0.17 events per year matching this peak) and thus the value of the covariate Max_PSD=0.17 in this case. Note that the adjacent (second highest) power value of approximately 0.16 is also significant based on the abovementioned hypothesis; however, its magnitude is similar and thus redundant in interpretation with the highest PSD value. For such instances, we made an assumption that significant PSDs with adjacent frequency values should be treated as part of the same "cluster" of PSD peaks and counted the number of such clusters in the periodogram outputs rather than significant PSD values themselves to generate the variable characterizing the number of pronounced periodic events in the series (NumPeaksPSD). In this Figure's example, the number of such clusters equals to 4.

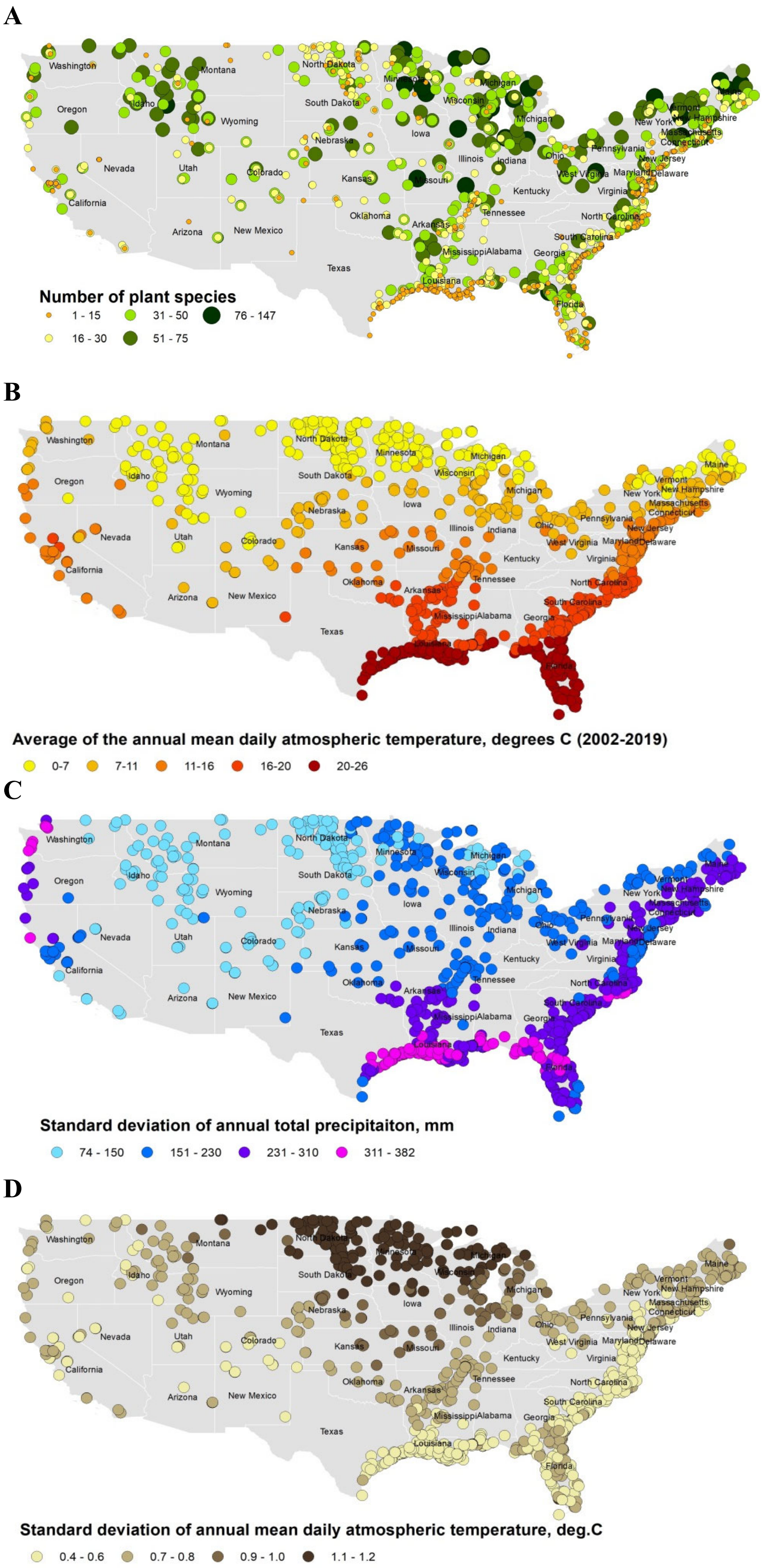
A
Number of plant species
1 - 15
16 - 30
31 - 50
51 - 75
76 - 147
B
Average of the annual mean daily atmospheric temperature, degrees C (2002-2019)
0-7
7-11
11-16
16-20
20-26
C
Standard deviation of annual total precipitaiton, mm
74 - 150
151 - 230
231 - 310
311 - 382
D
Standard deviation of annual mean daily atmospheric temperature, deg.C
0.4 - 0.6
0.7 - 0.8
0.9 - 1.0
1.1 - 1.2

E

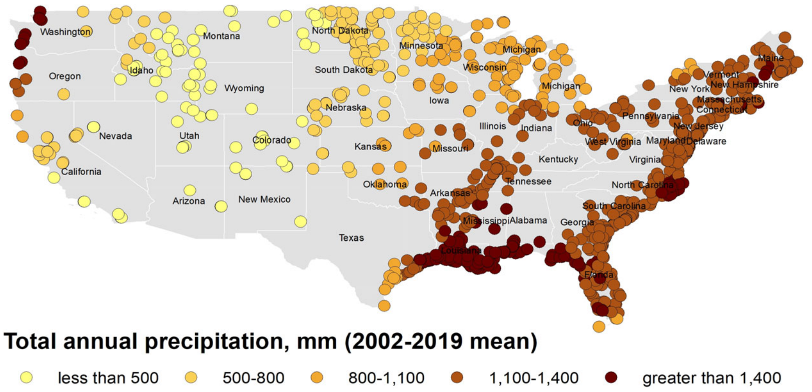

F

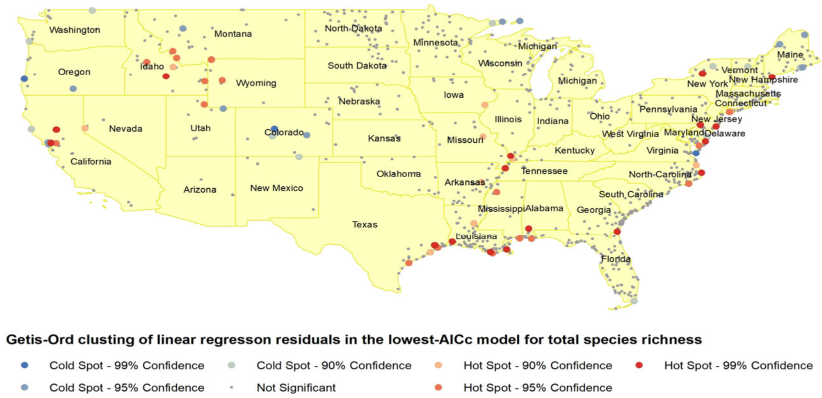

Fig. S3. Geographic distributions of selected variables: (A) Taxonomic diversity of vascular plants (here showing total species richness as example given high correlation between all five diversity metrics with Pearson's correlation coefficient ≥0.78 and p-value<0.01 for all pairs); (B) Average of the annual mean daily atmospheric temperature; (C) Standard deviation of the annual total precipitation; (D) Total annual precipitation; (E) Standard deviation of the annual mean daily atmospheric temperature; (F) Getis-Ord G_i statistic ***(54)*** for residuals of the highest-support (i.e., lowest value of Akaike Information Criterion, AIC_c) linear regression model with phenological variability as response variable and species richness and covariates as dependent variables. Getis-Ord G_i indicates wetland sites with significant spatial association of similar high values ("hot spots") or low values ("cold spots") estimated in ArcGIS Desktop v.10.8 software (Esri Inc.); wetland sites identified as significant hot and cold spots are more similar to their neighboring sites' values than expected by chance.

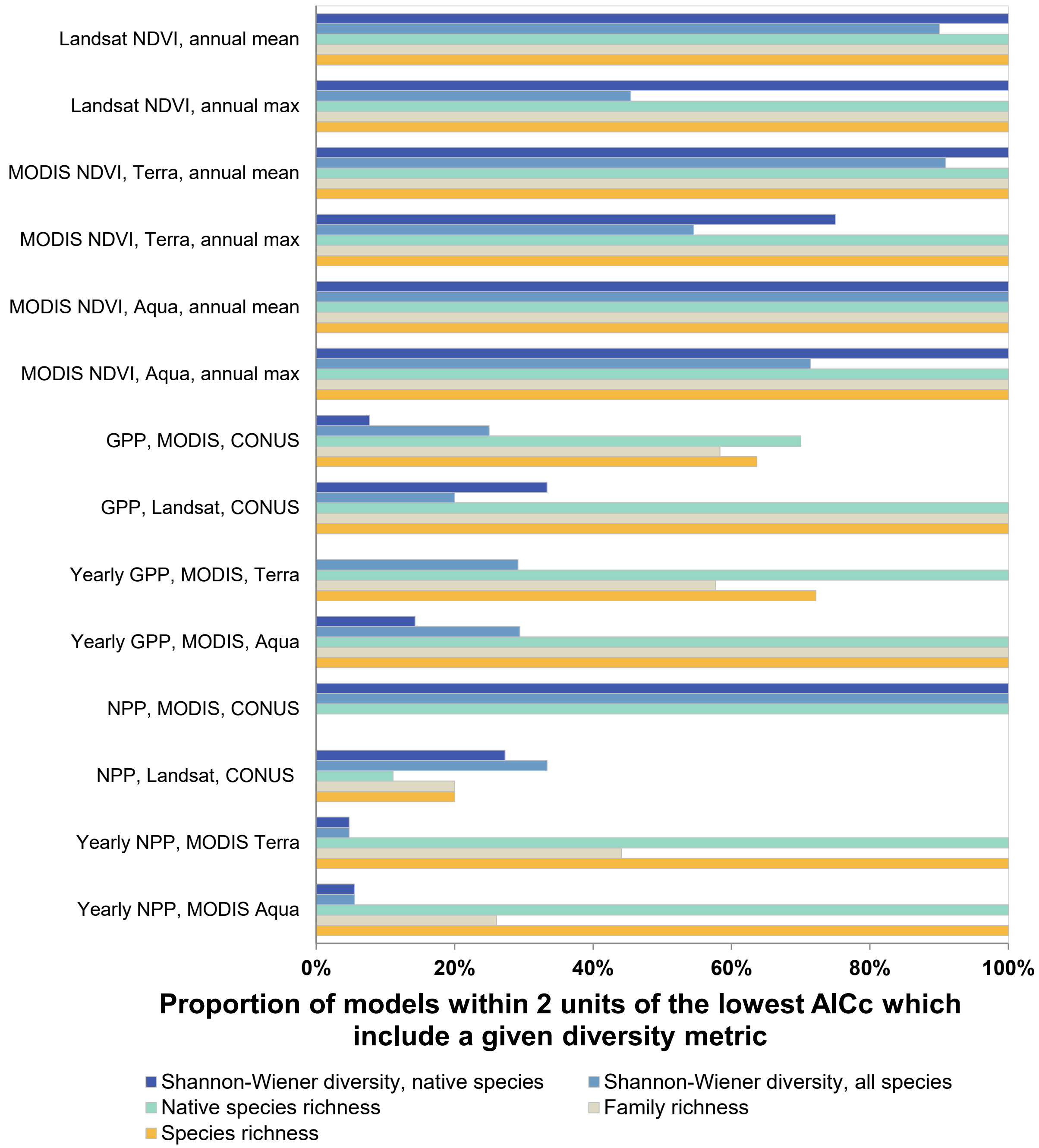

Fig. S4. Inclusion of plant diversity metrics in the linear multivariate regression models with the highest statistical support (i.e., models within two units of the model with the lowest value of Akaike Information Criterion, AIC_c) where dependent variables are stability indicators representing mean-to-standard deviation ratios in Normalized Difference Vegetation Index (annual mean and maxima), and gross and net primary productivity (GPP and NPP, respectively), derived from MODIS and Landsat satellite products.

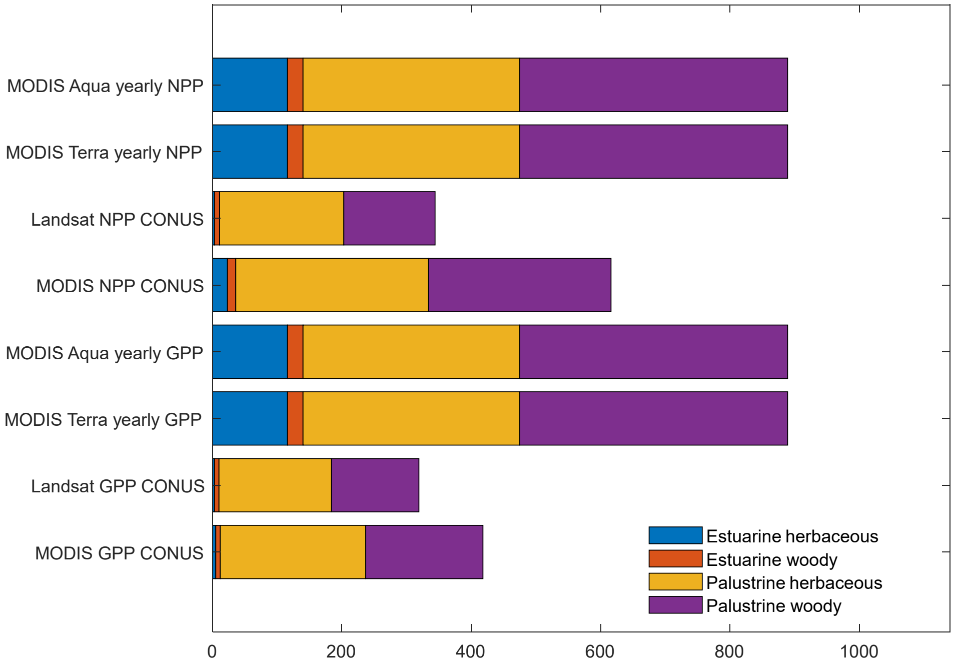

Figure S5. Representation of major wetland types based on the sites sampled by 2011 National Wetland Condition Assessment (NWCA) in spatial footprint of selected gridded Landsat and MODIS satellite-based gross and net primary productivity spatial datasets.

Table S1. Metrics of vascular plant diversity from the National Wetland Condition Assessment (NWCA) dataset (*39*, *63*)**.** All metrics listed in this table were derived from the survey of vascular plant species in a 500 m^2 sample of five field plots per wetland site.

**Metric**	**Definition**	**Statistical transformation for linear regression models, if any**
Total species richness (**TotSpecies_S**)	Total number of vascular plant species (based on TOTN_SPP in NWCA database)	Logarithmic transformation (base 10)
Native species richness (**NatSpecies_S**)	Total number of vascular plant species within the site designated as native to the site's geographic region (based on TOTN_NATSP in NWCA database)	Logarithmic transformation (base 10)
Total family richness (**Family_S**)	Total number of families representing vascular plant species within the site (based on TOTN_FAM in NWCA)	Logarithmic transformation (base 10)
Shannon-Wiener diversity index for all species (**TotSpecies_H**)	Negative sum of the products between each species' proportions of individuals relative to the total across all species and natural logarithms of these respective proportions (based on H_ALL in NWCA)	No transformation
Shannon-Wiener diversity index for native species (**NatSpecies_H**)	Negative sum of the products between each species' proportions of individuals relative to the total across species designated to be native to the site's geographic region and natural logarithms of these respective proportions (based on H_NAT in NWCA)	No transformation

Table S2. Candidate covariates of diversity metrics tested as explanatory variables in regression models for phenological variability as the response.

Category	Name	Description	Source
Climate, due to potential effects on vegetation green-up & senescence (*5*, *12*, *44*)	**MeanMeanTemp**	Average of the annual mean daily atmospheric temperature, 2002-2019	PRISM Daily Spatial Climate Dataset AN81d (Parameter-elevation Regressions on Independent Slopes Model) daily via Google Earth Engine (*51*), spatial resolution 2.5 arc minutes
	**StdevMeanTemp**	Standard deviation of the annual mean daily atmospheric temperature, 2002-2019	
	**MeanSumPrecip**	Average of the total annual precipitation, 2002-2019	Precipitation Estimation from Remotely Sensed Information using Artificial Neural Networks- Climate Data Record (PERSIANN-CDR) via Google Earth Engine (*51*), spatial resolution 0.25 arc degrees
	**StdevSumPrecip**	Standard deviation of the total annual precipitation, 2002-2019	
Variability in greenness that may result from spectral interplay among vegetation, non-vegetated wetland surfaces & flooding(*27*, *28*)	**Type_PH**	One of the four major NWCA wetland types, Palustrine herbaceous (1=palustrine herbaceous, 0=other wetland types)	NWCA 2011 survey (*39*, *63*). These three binary dummy variables represent four major NWCA wetland types defined by hydrology and predominant vegetation: palustrine woody (PW), palustrine herbaceous (PH), estuarine woody (EW) and estuarine herbaceous (EH). Only three binary variables are used in the models because together they automatically account for the fourth category.
	**Type_EW**	One of the four major NWCA wetland types, Estuarine woody (1=estuarine woody, 0=other wetland types)	
	**Type_EH**	One of the four major NWCA wetland types, Estuarine herbaceous (1=estuarine herbaceous, 0=other wetland types)	
	**Max_PSD**	Maximum value of the power density spectrum of the dominant cycle in 2002-2019 greenness time series, estimated by a periodogram analysis	Greenness approximated by 8-day, 250m-spatial resolution 2002-2019 time series of the Normalized Difference Vegetation Index (NDVI) computed from MOD09Q1.006 product from the Moderate Resolution Imaging Spectroradiometer (Terra satellite)
	**NumPeaksPSD**	Number of periodic cycle series detected within 2002-2019 greenness time series with significantly high values of the power density spectrum estimated by a periodogram analysis	
	**Var_LST**	Inter-annual variability in seasonal land surface temperature (LST) computed as an average deviation of LST values of the seasonal 8-day blocks from their respective means over 2002-2009	MOD11A2.006 Terra Land Surface Temperature and Emissivity 8-Day Global 1km product via Google Earth Engine, gap-filled as discussed in Materials & Methods
Vegetation & site characteristics potentially related to magnitude & variability in greenness	**MedTallVeg_Freq**	Combined frequency of medium and tall vegetation as the sum of NWCA variables for specific height classes (FREQ_MED_V for 2-5m tall, FREQ_HMED for 5-15m tall, FREQ_TALL for 15-30m tall, FREQ_VTALL for >30m tall).	NWCA 2011 survey (*39*, *63*)
	**Soil_pH**	Mean soil pH across five sampling plots of each wetland site's assessment area	NWCA 2011 survey (*39*, *63*)
Disturbance proxies	**Disturbance**	Disturbance and stress status based on disturbance score assigned by NWCA (REF_NWCA variable), converted from three original levels (least, intermediate & most disturbance) to binary (0=least disturbed, 1=intermediately or most disturbed).	NWCA 2011 survey (*39*, *63*)
	**Agric_Severity**	Cumulative severity of agricultural stressors in the wetland's buffer zone, defined as the land adjacent to the assessment area with maximum width of 100m (AGR_SEVERITY_M3 in NWCA). Here four levels of the original variable (scores of 0,1,2,3 with no stressors equal to 0 and maximum stressors equal to 3) were converted to a binary variable where 0=no stressors (original score of 0) and 1=any stress level (original score 1, 2 or 3).	NWCA 2011 survey (*39*, *63*)

Table S3. Standardized regression coefficients and their respective standard errors (in parentheses) for explanatory variables selected in the highest-support model (i.e., lowest-AIC$_c$ where AICc stands for Akaike Information Criterion (*40*) corrected for the number of estimated parameters) model for three indicators of stability (i.e., phenological variability and mean-to-standard deviation ratios in annual maximum and annual mean NDVI) derived from the same MOD09Q1.006 satellite product. Abbreviated variables ae defined in Table S2. Other covariates introduced in Table S2 were tested but not always included in the highest-support models. Mean atmospheric temperature (MeanMeanTemp) was transformed as a second-order polynomial in regression models, and its standardized coefficient here is provided for the quadratic term. ***p-value≤0.001, **0.001<p-value≤0.01, *0.01<p-value≤0.05

Explanatory variable:	Plant diversity metric				
	**Total species richness (log)**	**Family richness (log)**	**Native species richness (log)**	**Shannon-Wiener index, all species**	**Shannon-Wiener index, native species**
Dependent variable: Phenological variability					
Plant diversity metric (columns)	**-0.131*** (0.024)**	**-0.129*** (0.024)**	**-0.133*** (0.024)**	**-0.089*** (0.023)**	**-0.105*** (0.023)**
MeanMeanTemp	**0.839*** (0.093)**	**0.828*** (0.093)**	**0.866*** (0.093)**	**0.856*** (0.093)**	**0.868*** (0.094)**
StdevMeanTemp	**0.114*** (0.025)**	**0.116*** (0.025)**	**0.118*** (0.025)**	**0.110*** (0.026)**	**0.112***(0.026)**
StdevSumPrecip	**-0.086** (0.029)**	**-0.086** (0.029)**	**-0.080** (0.029)**	**-0.089** (0.029)**	**-0.083** (0.029)**
Soil_pH	**0.189*** (0.189)**	**0.188*** (0.022)**	**0.185*** (0.022)**	**0.189*** (0.022)**	**0.185*** (0.022)**
Max_PSD	**0.291*** (0.019)**	**0.292*** (0.019)**	**0.290*** (0.019)**	**0.298*** (0.019)**	**0.297*** (0.019)**
NumPeaksPSD	**-0.040* (0.019)**	**-0.041* (0.019)**	**-0.037* (0.019)**	**-0.041* (0.019)**	**-0.039* (0.019)**
MedTallVeg_Freq	**-0.141*** (0.022)**	**-0.132*** (0.023)**	**-0.137*** (0.022)**	**-0.156*** (0.022)**	**-0.147*** (0.022)**
Var_LST	**-0.052** (0.019)**	**-0.051** (0.019)**	**-0.051** (0.019)**	**-0.055** (0.019)**	**-0.055** (0.019)**
Agric_Severity	**0.338*** (0.044)**	**0.336*** (0.044)**	**0.334*** (0.044)**	**0.322***(0.044)**	**0.321*** (0.044)**
Model adjusted R^2	0.662	0.662	0.665	0.658	0.660
Dependent variable: Stability (mean to standard deviation ratio of annual maximum greenness					
Plant diversity metric (columns)	**0.105*** (0.034)**	**0.137*** (0.034)**	**0.121*** (0.032)**	0.049 (0.030)	0.056 (0.030)
MeanMeanTemp	**-0.372** (0.117)**	**-0.359** (0.117)**	**-0.389** (0.117)**	**-0.381** (0.118)**	**-0.388** (0.118)**
StdevMeanTemp	**0.185*** (0.031)**	**0.188*** (0.031)**	**0.185*** (0.031)**	**0.183*** (0.031)**	**0.183*** (0.031)**
Soil_pH	**-0.086** (0.027)**	**-0.088** (0.027)**	**-0.083** (0.027)**	**-0.085** (0.027)**	**-0.083** (0.027)**
Max_PSD	**-0.056* (0.023)**	**-0.055* (0.023)**	**-0.057* (0.023)**	**-0.061** (0.023)**	**-0.061** (0.023)**
MedTallVeg_Freq	**0.257*** (0.027)**	**0.240*** (0.028)**	**0.248*** (0.028)**	**0.268*** (0.028)**	**0.265*** (0.028)**
Var_LST	-0.023 (0.024)	-0.024 (0.024)	-0.021 (0.024)	-0.022 (0.024)	-0.022 (0.024)
Agric_Severity	**-0.312*** (0.056)**	**-0.310*** (0.056)**	**-0.314*** (0.056)**	**-0.311*** (0.056)**	**-0.310*** (0.056)**
Type_EW	**-0.606*** (0.098)**	**-0.594*** (0.097)**	**-0.609*** (0.097)**	**-0.618*** (0.098)**	**-0.622*** (0.098)**
Type_EH	**-0.542*** (0.079)**	**-0.495*** (0.081)**	**-0.535*** (0.077)**	**-0.609*** (0.076)**	**-0.608*** (0.075)**
Model adjusted R^2	0.503	0.506	0.506	0.500	0.500
Dependent variable: Stability (mean to standard deviation ratio of annual mean greenness					
Plant diversity metric (columns)	**0.124*** (0.033)**	**0.151*** (0.034)**	**0.137*** (0.033)**	Not included	**-0.065* (0.030)**
MeanMeanTemp	**-0.419***(0.114)**	**-0.404***(0.114)**	**-0.434***(0.114)**	**-0.432***(0.115)**	**-0.441*** (0.115)**
MeanSumPrecip	**0.315***(0.031)**	**0.308*** (0.031)**	**0.305*** (0.031)**	**0.324***(0.031)**	**0.315*** (0.031)**
Disturbance	**0.111* (0.053)**	**0.112* (0.053)**	0.103 (0.053)	0.092 (0.053)	0.094 (0.053)
NumPeaksPSD	-0.039 (0.023)	-0.039 (0.023)	-0.041 (0.023)	-0.036 (0.023)	-0.039* (0.019)
MedTallVeg_Freq	**0.395*** (0.034)**	**0.379*** (0.035)**	**0.388*** (0.034)**	**0.422*** (0.034)**	**0.403*** (0.035)**
Var_LST	-0.016 (0.024)	-0.017 (0.024)	-0.016 (0.024)	-0.014 (0.024)	-0.015 (0.024)
Agric_Severity	**-0.152**(0.055)**	**-0.150*** (0.055)**	**-0.149**(0.055)**	**-0.147**(0.056)**	**-0.146** (0.056)**
Type_PH	**-0.243**(0.070)**	**-0.242** (0.070)**	**-0.230** (0.070)**	**-0.262** (0.070)**	**-0.243**(0.071)**
Type_EW	**-0.262* (0.103)**	**-0.250* (0.103)**	**-0.265* (0.103)**	**-0.298** (0.103)**	**-0.281** (0.104)**
Type_EH	**-0.390*** (0.096)**	**-0.347*** (0.097)**	**-0.383*** (0.095)**	**-0.542*** (0.087)**	**-0.469*** (0.093)**
Model adjusted R^2	0.500	0.503	0.502	0.495	0.496

Table S4. Alternative indicators of stability derived from satellite data, and their correlations with phenological consistency metric developed in this study.

Variable name	Input satellite product	Grid cell size, m	Number of 2011 NWCA wetland sites covered by the product	Stability measure	Adjusted R^2 of the lowest-AIC_c model with diversity & covariates for a given stability measure				
					TotSpecies_S	NatSpecies_S	Family_S	TotSpecies_H	NatSpecies_H
MSDR_Landsat_Mean	Normalized Difference Vegetation Index (NDVI) computed from Landsat 5,7,8 Tier-1 surface reflectance	30	1138	Mean-to-standard deviation ratio of site-average annual mean NDVI	0.304	0.304	0.303	0.296	0.297
MSDR_Landsat_Max		30	1138	Mean-to-standard deviation ratio of site-average annual maximum NDVI	0.276	0.280	0.276	0.272	0.274
MSDR_ModTerra_8d_AnnMean	MODIS Terra 8-day NDVI computed from MOD09Q1.006 Terra Surface Reflectance 8-Day Global 250m	250	1138	Mean-to-standard deviation ratio of site-average annual mean NDVI	0.500	0.502	0.503	0.495	0.496
MSDR_ModTerra_8d_AnnMax		250	1138	Mean-to-standard deviation ratio of site-average annual maximum NDVI	0.503	0.506	0.506	0.500	0.500
MSDR_ModAqua_8d_AnnMean	MODIS Aqua 8-day NDVI MYD09Q1.006 Aqua Surface Reflectance 8-Day Global 250m	250	1138	Mean-to-standard deviation ratio of site-average annual mean NDVI	0.313	0.314	0.312	0.309	0.311
MSDR_ModAqua_8d_AnnMax		250	1138	Mean-to-standard deviation ratio of site-average annual maximum NDVI	0.504	2.272	0.505	0.499	0.500
MSDR_MODIS_CONUS_GPP	Annual mean gross primary production (GPP) from CONUS product, MODIS (*64*)	250	616	Mean-to-standard deviation ratio of annual mean values	0.370	0.370	0.369	0.368	0.368
MSDR_Landsat_CONUS_GPP	Annual mean GPP from CONUS product, Landsat (*64*)	30	344	Site-average mean-to-standard deviation ratio of annual mean values	0.315	0.318	0.318	0.301	0.301
MSDR_MODIS_8d_Terra_GPP	Annual mean GPP computed from MOD17A2H.006: Terra Gross Primary Productivity 8-Day Global 500M	500	889	Site-average mean-to-standard deviation ratio of annual mean values	0.255	0.257	0.254	0.252	0.252
MSDR_MODIS_8d_Aqua_GPP	Annual mean GPP computed from MYD17A2H.006: Aqua Gross Primary Productivity 8-Day Global 500M	500	889	Site-average mean-to-standard deviation ratio of annual mean values	0.189	0.189	0.188	0.184	0.184
MSDR_MODIS_CONUS_NPP	Annual mean net primary production (NPP) from CONUS product, MODIS (*64*)	250	616	Site-average mean-to-standard deviation ratio of annual mean values	0.414	0.413	0.414	0.420	0.418
MSDR_Landsat_CONUS_NPP	Annual mean NPP from CONUS product, Landsat (*64*)	30	344	Site-average mean-to-standard deviation ratio of annual mean values	0.253	0.253	0.253	0.253	0.253
MSDR_MODIS_Terra_NPP	MOD17A3H.006: Terra Net Primary Production Yearly Global 500m	500	889	Site-average mean-to-standard deviation ratio of annual product values	0.040	0.039	0.038	0.036	0.036
MSDR_MODIS_Aqua_NPP	MYD17A3H.006: Aqua Net Primary Production Yearly Global 500m	500	889	Site-average mean-to-standard deviation ratio of annual product values	0.085	0.084	0.082	0.082	0.082

Table S5. Pearson's correlation coefficients for plant diversity, covariates, and indicators of stability. Top right corner portion of the table shows correlations among five diversity metrics (Table S1) and continuous covariates (Table S2), while bottom left portion indicates correlations among stability measures computed as mean-to-standard deviation ratios of satellite annual Normalized Difference Vegetation Index (NDVI), net primary productivity (NPP) and gross primary productivity (GPP) described in Table S4. **0.001<p-value≤0.01, *0.01<p-value≤0.05.

	TotSpecies_S	Family_S	NatSpecies_S	TotSpecies_H	NatSpecies_H	MeanMeanTemp	StdevMeanTemp	MeanSumPrecip	StdevSumPrecip	Soil_pH	MedTallVeg_Freq	Max_PSD	Var_LST	PhenoVariability	
MSDR_Landsat_Mean		.971**	.981**	.860**	.842**	-.448**	.289**	-.225**	-.298**	0.019	.486**	.427**	.250**	0.009	TotSpecies_S
MSDR_Landsat_Max	.488**		.959**	.843**	.834**	-.350**	.226**	-.133**	-.215**	-0.030	.567**	.374**	.230**	-.070*	Family_S
MSDR_ModTerra_8d_AnnMean	.521**	.407**		.861**	.871**	-.411**	.261**	-.150**	-.237**	-0.041	.523**	.413**	.229**	-0.017	NatSpecies_S
MSDR_ModTerra_8d_AnnMax	.260**	.647**	.419**		.957**	-.293**	.151**	-.112**	-.163**	-0.037	.546**	.278**	.199**	-.063*	TotSpecies_H
MSDR_ModAqua_8d_AnnMean	.542**	.332**	.838**	.336**		-.278**	.141**	-0.055	-.123**	-.095**	.565**	.280**	.184**	-.089**	NatSpecies_H
MSDR_ModAqua_8d_AnnMax	.251**	.655**	.397**	.839**	.302**		-.668**	.651**	.750**	-.200**	0.026	-.754**	-.305**	-.550**	MeanMeanTemp
MSDR_MODIS_CONUS_GPP	.426**	.391**	.504**	.435**	.442**	.417**		-.502**	-.576**	.390**	-0.031	.713**	.302**	.522**	StdevMeanTemp
MSDR_Landsat_CONUS_GPP	.591**	.415**	.439**	.316**	.361**	.285**	.671**		.893**	-.476**	.187**	-.393**	-.297**	-.467**	MeanSumPrecip
MSDR_MODIS_8d_Terra_GPP	.256**	.260**	.261**	.251**	.176**	.270**	.572**	.471**		-.371**	.091**	-.549**	-.298**	-.469**	StdevSumPrecip
MSDR_MODIS_8d_Aqua_GPP	.363**	.263**	.391**	.186**	.370**	.236**	.553**	.446**	.625**		-.259**	.200**	.132**	.425**	Soil_pH
MSDR_MODIS_CONUS_NPP	.315**	.312**	.266**	.345**	.230**	.318**	.743**	.572**	.540**	.451**		.077**	.069*	-.296**	MetTallVeg_Freq
MSDR_Landsat_CONUS_NPP	.335**	.246**	.184**	.162**	0.093	.181**	.462**	.667**	.459**	.397**	.599**		.281**	.574**	Max_PSD
MSDR_MODIS_Terra_NPP	0.026	-.074*	-0.058	-.116**	-0.032	-.069*	.111*	-0.008	.108**	.150**	.149**	0.037		.150**	Var_LST
MSDR_MODIS_Aqua_NPP	.094**	-0.007	-0.031	-.067*	-0.047	0.008	.260**	0.104	.182**	.259**	.263**	.203**	.715**		PhenoVariability
	MSDR_Landsat_Mean	MSDR_Landsat_Max	MSDR_ModTerra_8d_AnnMean	MSDR_ModTerra_8d_AnnMax	MSDR_ModAqua_8d_AnnMean	MSDR_ModAqua_8d_AnnMax	MSDR_MODIS_CONUS_GPP	MSDR_Landsat_CONUS_GPP	MSDR_MODIS_8d_Terra_GPP	MSDR_MODIS_8d_Aqua_GPP	MSDR_MODIS_CONUS_NPP	MSDR_Landsat_CONUS_NPP	MSDR_MODIS_Terra_NPP	MSDR_MODIS_Aqua_NPP	

Table S6. Standardized regression weights of the structural equation models (SEMs) (*60*). For all models shown, the probability of their respective chi-squared (χ^2) was >0.1, indicating no evidence that hypotheses tested by SEMs were different from data structure. PhenoVariability is phenological variability; Stability is mean-to-standard deviation ratio of annual mean NDVI computed from the same satellite product (MOD09Q1.006); df is degrees of freedom; CFI is comparative fit index; RMSE is root mean square error. Alternative models that did not meet diagnostic criteria (χ^2:df <3; CFI>0.95) are not reported. Weights in parentheses next to stability in column 2 represent direct path from phenological variability to stability. ***p-value≤0.001, **0.001<p-value≤0.01, *0.01<p-value≤0.05.

**Diversity metric**	**Path from variables to:**	**Diversity**	**MeanMean Temp**	**StdevMean Temp**	**MeanSum Precip**	**StdevSum Precip**	**Soil pH**	**MedTallVeg Freq**	**Agric Severity**	**Max PSD**	**Var LST**	**χ2**	**χ2:df ratio**	**CFI**	**RMSE**
**TotSpecies_S**	PhenoVariability	-0.231***	-0.282***	-0.001	-0.214***	0.201***	0.228***	-0.127***	0.022	0.465***	-0.039	1.551	1.551	1.000	0.022
	Stability (-0.657***)	0.056	-0.108*	-0.001	0.038	0.056	-0.029	0.150***	n/a	0.149	0.017				
**TotSpecies_S**	PhenoVariability	-0.236***	-0.277***	n/a	-0.218***	0.204***	0.225***	-0.127***	n/a	0.468***	-0.038	3.362	1.681	1.000	0.024
	Stability (-0.657***)	n/a	-0.277***	n/a	0.041	0.062	n/a	0.176***	n/a	0.169***	0.022				
**TotSpecies_S**	PhenoVariability	-0.241***	-0.276***	n/a	-0.208***	0.201***	0.224***	-0.129***	n/a	0.463***	n/a	3.553	1.776	1.000	0.026
	Stability (-0.658***)	n/a	-0.146***	n/a	0.034	0.064	n/a	0.178***	n/a	0.174***	n/a				
**TotSpecies_S**	PhenoVariability	-0.241***	-0.279***	n/a	-0.208***	0.201***	0.224***	-0.129***	n/a	0.463***	n/a	3.957	1.319	1.000	0.017
	Stability (-0.662***)	n/a	-0.144***	n/a	n/a	0.095**	n/a	0.179***	n/a	0.181***	n/a				
**Family_S**	PhenoVariability	-0.244***	-0.260***	-0.003	-0.204***	0.195***	0.227***	-0.104***	0.022	0.472***	-0.037	1.543	1.543	1.000	0.022
	Stability (-0.635***)	0.059	-0.113*	0.025	0.036	0.058	-0.029	0.144***	n/a	0.146***	0.017				
**Family_S**	PhenoVariability	-0.249***	-0.254***	n/a	-0.209***	0.197***	0.224***	-0.103***	n/a	0.475***	-0.036	3.664	1.832	1.000	0.027
	Stability (-0.656***)	n/a	-0.145***	n/a	0.041	0.062	n/a	0.176***	n/a	0.169**	0.022				
**Family_S**	PhenoVariability	-0.253***	-0.253***	n/a	-0.199***	0.195***	0.224***	-0.104***	n/a	0.470***	n/a	3.892	1.946	1.000	0.029
	Stability (-0.658***)	n/a	-0.146***	n/a	0.034	0.064	n/a	0.178***	n/a	0.174***	n/a				
**Family_S**	PhenoVariability	-0.253***	-0.253***	n/a	-0.199***	0.195***	0.224***	-0.104***	n/a	0.470***	n/a	4.297	1.432	1.000	0.019
	Stability (-0.662***)	n/a	-0.144***	n/a	n/a	0.095**	n/a	0.179***	n/a	0.181***	n/a				
**NatSpecies_S**	PhenoVariability	-0.212***	-0.277***	0.004	-0.198***	0.200***	0.221***	-0.132***	0.033	0.462***	0.042	1.453	1.453	1.000	0.020
	Stability (-0.633***)	0.076*	-0.100*	0.026	0.037	0.053	-0.029	0.139***	n/a	0.144***	0.016				
**NatSpecies_S**	PhenoVariability	-0.217***	-0.270***	n/a	-0.204***	0.203***	0.217***	-0.132***	n/a	0.470***	-0.040	0.670	0.670	1.000	<0.001
	Stability (-0.640***)	0.072*	-0.116*	n/a	0.051	0.049	n/a	0.143***	n/a	0.152***	0.017				
**NatSpecies_S**	PhenoVariability	-0.222***	-0.269***	n/a	-0.193***	0.200***	0.217***	-0.135***	n/a	0.464***	n/a	0.643	0.643	1.000	<0.001
	Stability (-0.641***)	0.074*	-0.116*	n/a	0.045	0.050	n/a	0.144***	n/a	0.155***	n/a				
**NatSpecies_S**	PhenoVariability	-0.222***	-0.269***	n/a	-0.193***	0.200***	0.217***	-0.135***	n/a	0.464***	n/a	1.347	0.674	1.000	<0.001
	Stability (-0.647***)	0.072*	-0.113*	n/a	n/a	0.091**	n/a	0.147***	n/a	0.166***	n/a				
**TotSpecies_H**	PhenoVariability	-0.132***	-0.240***	0.004	-0.204***	0.195***	0.226***	-0.166***	0.042*	0.438***	-0.048*	1.160	1.160	1.000	0.012
	Stability (-0.644***)	0.043	-0.117*	0.025	0.037	0.057	-0.027	0.151***	n/a	0.157***	0.018				
**TotSpecies_H**	PhenoVariability	-0.132***	-0.241***	n/a	-0.204***	0.195***	0.227***	-0.166***	0.042*	0.440***	-0.047*	3.562	1.187	1.000	0.013
	Stability (-0.656***)	n/a	-0.145***	n/a	0.041	0.062	n/a	0.176***	n/a	0.169***	0.021				
**TotSpecies_H**	PhenoVariability	-0.132***	-0.241***	n/a	-0.204***	0.195***	0.227***	-0.166***	0.042*	0.440***	-0.047*	4.347	1.087	1.000	0.009
	Stability (-0.658***)	n/a	-0.146***	n/a	0.034	0.064	n/a	0.178***	n/a	0.174***	n/a				
**TotSpecies_H**	PhenoVariability	-0.132***	-0.241***	n/a	-0.204***	0.195***	0.227	-0.166***	0.042*	0.440***	-0.047*	4.751	0.950	1.000	<0.001
	Stability (-0.662***)	n/a	-0.144***	n/a	n/a	0.095**	n/a	0.179***	n/a	0.181***	n/a				
**NatSpecies_H**	PhenoVariability	-0.142***	-0.245***	0.006	-0.195***	0.195***	0.221***	-0.161***	0.045*	0.439***	-0.048*	1.087	1.087	1.000	0.009
	Stability (-0.642***)	0.055	-0.112*	0.025	0.035	0.055	-0.026	0.146***	n/a	0.155***	0.017				
**NatSpecies_H**	PhenoVariability	-0.142***	-0.247***	n/a	-0.195***	0.194***	0.222***	-0.161***	0.045*	0.442***	-0.047*	4.701	1.567	1.000	0.022
	Stability (-0.656***)	n/a	-0.145***	n/a	0.041	0.062	n/a	0.176***	n/a	0.169***	0.021				
**NatSpecies_H**	PhenoVariability	-0.142***	-0.247***	n/a	-0.195***	0.194***	0.222***	-0.161***	0.045*	0.442***	-0.047*	5.483	1.371	1.000	0.018
	Stability (-0.658***)	n/a	-0.146***	n/a	0.034	0.064	n/a	0.178***	n/a	0.174***	n/a				
**NatSpecies_H**	PhenoVariability	-0.142***	-0.247***	n/a	-0.195***	0.194***	0.222***	-0.161***	0.045*	0.442***	-0.047*	5.888	1.178	1.000	0.012
	Stability (-00.662***)	n/a	-0.144***	n/a	n/a	0.095**	n/a	0.179***	n/a	0.181***	n/a				

Other Supplementary Materials: Data files and computing codes

The following datasets associated with our study analyses can be downloaded from the Dryad repository "Auxiliary supplementary material for the study on plant diversity relationships with satellite-detected phenological variability in USA wetlands at a national scale", located at https://doi.org/10.6078/D13T40 and https://zenodo.org/record/6774517#.YsxlnnbMIuU:

S1: Data_S1_Regression_Dataset_v4_22_2022.xslx: plant diversity and wetland site variables for 1138 wetland sites used in this study's statistical analyses.

S2: Data_S2_Code_for_Custom_Variables.zip: a Zip file containing a README explanatory text file, two Matlab codes for estimating measures of phenological variability and its two custom-derived covariates and two input data files required to run the codes: the dataset original 2002-2019 series of Normalized Difference Vegetation Index computed from wetland sites for MOD09Q1.006 product and the dataset with site names.

S3: Data_S3_Code_for_Variability_LST.zip: a Zip file containing a README_LST explanatory text file, two Matlab codes for estimating variability in land surface temperature (LST) from MOD11A2.006 Terra Land Surface Temperature and Emissivity 8-Day Global 1km product and required input files.

REFERENCES AND NOTES

1. M. Loreau, C. de Mazancourt, Biodiversity and ecosystem stability: A synthesis of underlying mechanisms. *Ecol. Lett.* **16**, 106–115 (2013).

2. S. Yachi, M. Loreau, Biodiversity and ecosystem productivity in a fluctuating environment: The insurance hypothesis. *Proc. Natl. Acad. Sci. U.S.A.* **96**, 1463–1468 (1999).

3. M. Loreau, N. Mouquet, A. Gonzalez, Biodiversity as spatial insurance in heterogeneous landscapes. *Proc. Natl. Acad. Sci. U.S.A.* **100**, 12765–12770 (2003).

4. G. G. Mazzochini, C. R. Fonseca, G. C. Costa, R. M. Santos, A. T. Oliveira-Filho, G. Ganade, Plant phylogenetic diversity stabilizes large-scale ecosystem productivity. *Glob. Ecol. Biogeogr.* **28**, 1430–1439 (2019).

5. P. García-Palacios, N. Gross, J. Gaitán, F. T. Maestre, Climate mediates the biodiversity-ecosystem stability relationship globally. *Proc. Natl. Acad. Sci. U.S.A.* **115**, 8400–8405 (2018).

6. Y. Hautier, D. Tilman, F. Isbell, E. W. Seabloom, E. T. Borer, P. B. Reich, Anthropogenic environmental changes affect ecosystem stability via biodiversity. *Science* **348**, 336–340 (2015).

7. C. R. Zirbel, E. Grman, T. Bassett, L. A. Brudvig, Landscape context explains ecosystem multifunctionality in restored grasslands better than plant diversity. *Ecology* **100**, e02634 (2019).

8. F. van der Plas, Biodiversity and ecosystem functioning in naturally assembled communities. *Biol. Rev.* **94**, 1220–1245 (2019).

9. B. J. Cardinale, J. E. Duffy, A. Gonzalez, D. U. Hooper, C. Perrings, P. Venail, A. Narwani, G. M. Mace, D. Tilman, D. A. Wardle, A. P. Kinzig, G. C. Daily, M. Loreau, J. B. Grace, A. Larigauderie, D. S. Srivastava, S. Naeem, Biodiversity loss and its impact on humanity. *Nature* **486**, 59–67 (2012).

10. A. Gonzalez, R. M. Germain, D. S. Srivastava, E. Filotas, L. E. Dee, D. Gravel, P. L. Thompson, F. Isbell, S. Wang, S. Kéfi, J. Montoya, Y. R. Zelnik, M. Loreau, Scaling-up biodiversity-ecosystem functioning research. *Ecol. Lett.* **23**, 757–776 (2020).

11. A. S. Mori, T. Furukawa, T. Sasaki, Response diversity determines the resilience of ecosystems to environmental change. *Biol. Rev.* **88**, 349–364 (2013).

12. J. Oehri, B. Schmid, G. Schaepman-Strub, P. A. Niklaus, Biodiversity promotes primary productivity and growing season lengthening at the landscape scale. *Proc. Natl. Acad. Sci. U.S.A.* **114**, 10160–10165 (2017).

13. B.-G. J. Brooks, D. C. Lee, L. Y. Pomara, W. W. Hargrove, Monitoring broadscale vegetational diversity and change across North American landscapes using land surface phenology. *Forests* **11**, 606 (2020).

14. G. Rheault, E. Lévesque, R. Proulx, Diversity of plant assemblages dampens the variability of the growing season phenology in wetland landscapes. *BMC Ecol. Evol.* **21,** 91 (2021).

15. X. Morin, L. Fahse, C. de Mazancourt, M. Scherer-Lorenzen, H. Bugmann, Temporal stability in forest productivity increases with tree diversity due to asynchrony in species dynamics. *Ecol. Lett.* **17**, 1526–1535 (2014).

16. A. Viña, W. Liu, S. Zhou, J. Huang, J. Liu, Land surface phenology as an indicator of biodiversity patterns. *Ecol. Indic.* **64**, 281–288 (2016).

17. J. M. Samplonius, A. Atkinson, C. Hassall, K. Keogan, S. J. Thackeray, J. J. Assmann, M. D. Burgess, J. Johansson, K. H. Macphie, J. W. Pearce-Higgins, E. G. Simmonds, Ø. Varpe, J. C. Weir, D. Z. Childs, E. F. Cole, F. Daunt, T. Hart, O. T. Lewis, N. Pettorelli, B. C. Sheldon, A. B. Phillimore, Strengthening the evidence base for temperature-mediated phenological asynchrony and its impacts. *Nat. Ecol. Evol.* **5**, 155–164 (2021).

18. Z. Ma, H. Liu, Z. Mi, Z. Zhang, Y. Wang, W. Xu, L. Jiang, J.-S. He, Climate warming reduces the temporal stability of plant community biomass production. *Nat. Commun.* **8**, 15378 (2017).

19. A. K. Skidmore, N. C. Coops, E. Neinavaz, A. Ali, M. E. Schaepman, M. Paganini, W. D. Kissling, P. Vihervaara, R. Darvishzadeh, H. Feilhauer, M. Fernandez, N. Fernández, N. Gorelick, I. Geijzendorffer, U. Heiden, M. Heurich, D. Hobern, S. Holzwarth, F. E. Muller-Karger, R. Van De Kerchove, A. Lausch, P. J. Leitão, M. C. Lock, C. A. Mücher, B. O'Connor, D. Rocchini, C. Roeoesli,

W. Turner, J. K. Vis, T. Wang, M. Wegmann, V. Wingate, Priority list of biodiversity metrics to observe from space. *Nat. Ecol. Evol.* **5**, 896–906 (2021).

20. E. Grman, J. A. Lau, D. R. Schoolmaster, K. L. Gross, Mechanisms contributing to stability in ecosystem function depend on the environmental context. *Ecol. Lett.* **13**, 1400–1410 (2010).

21. L. M. Thibaut, S. R. Connolly, Understanding diversity-stability relationships: Towards a unified model of portfolio effects. *Ecol. Lett.* **16**, 140–150 (2013).

22. R. Wang, J. A. Gamon, Remote sensing of terrestrial plant biodiversity. *Remote Sens. Environ.* **231**, 111218 (2019).

23. J. Doležal, V. Lanta, O. Mudrák, J. Lepš, Seasonality promotes grassland diversity: Interactions with mowing, fertilization and removal of dominant species. *J. Ecol.* **107**, 203–215 (2019).

24. D. E. Rothstein, D. R. Zak, Photosynthetic adaptation and acclimation to exploit seasonal periods of direct irradiance in three temperate, deciduous-forest herbs. *Funct. Ecol.* **15**, 722–731 (2001).

25. L. M. Hallett, J. S. Hsu, E. E. Cleland, S. L. Collins, T. L. Dickson, E. C. Farrer, L. A. Gherardi, K. L. Gross, R. J. Hobbs, L. Turnbull, K. N. Suding, Biotic mechanisms of community stability shift along a precipitation gradient. *Ecology* **95**, 1693–1700 (2014).

26. M. Hisano, H. Y. H. Chen, Spatial variation in climate modifies effects of functional diversity on biomass dynamics in natural forests across Canada. *Glob. Ecol. Biogeogr.* **29**, 682–695 (2020).

27. S. Taddeo, I. Dronova, N. Depsky, Spectral vegetation indices of wetland greenness: Responses to vegetation structure, composition, and spatial distribution. *Remote Sens. Environ.* **234**, 111467 (2019).

28. M. S. Kearney, D. Stutzer, K. Turpie, J. C. Stevenson, The effects of tidal inundation on the reflectance characteristics of coastal marsh vegetation. *J. Coast. Res.* **25**, 1177–1186 (2009).

29. G. J. Miller, I. Dronova, P. Y. Oikawa, S. H. Knox, L. Windham-Myers, J. Shahan, E. Stuart-Haëntjens, The potential of satellite remote sensing time series to uncover wetland phenology under unique challenges of tidal setting. *Remote Sens. (Basel)* **13**, 3589 (2021).

30. J. M. Doherty, J. C. Callaway, J. B. Zedler, Diversity-function relationships changed in a long-term restoration experiment. *Ecol. Appl.* **21**, 2143–2155 (2011).

31. G. Sullivan, J. C. Callaway, J. B. Zedler, Plant assemblage composition explains and predicts how biodiversity affects salt marsh functioning. *Ecol. Monogr.* **77**, 569–590 (2007).

32. S. Taddeo, I. Dronova, K. Harris, The potential of satellite greenness to predict plant diversity among wetland types, ecoregions, and disturbance levels. *Ecol. Appl.* **29**, e01961 (2019).

33. H. Moor, H. Rydin, K. Hylander, M. B. Nilsson, R. Lindborg, J. Norberg, Towards a trait-based ecology of wetland vegetation. *J. Ecol.* **105**, 1623–1635 (2017).

34. J. B. Zedler, S. Kercher, Wetland resources: Status, trends, ecosystem services, and restorability. *Annu. Rev. Env. Resour.* **30**, 39–74 (2005).

35. W. J. Mitsch, B. Bernal, A. M. Nahlik, U. Mander, L. Zhang, C. J. Anderson, S. E. Jorgensen, H. Brix, Wetlands, carbon, and climate change. *Landsc. Ecol.* **28**, 583–597 (2013).

36. N. C. Davidson, How much wetland has the world lost? Long-term and recent trends in global wetland area. *Mar. Freshw. Res.* **65**, 934–941 (2014).

37. J. P. Gibbs, Wetland loss and biodiversity conservation. *Conserv. Biol.* **14**, 314–317 (2000).

38. S. Hu, Z. Niu, Y. Chen, L. Li, H. Zhang, Global wetlands: Potential distribution, wetland loss, and status. *Sci. Total Environ.* **586**, 319–327 (2017).

39. U.S. Environmental Protection Agency (EPA), "National Wetland Condition Assessment 2011 Technical Report" (Technical Report EPA-843-R-15-006, Washington, DC, 2016); www.epa.gov/national-aquatic-resource-surveys/national-wetland-condition-assessment-2011-technical-report.

40. H. Akaike, A new look at the statistical model identification. *IEEE Trans. Automat. Contr.* **19**, 716–723 (1974).

41. B. L. Bedford, The need to define hydrologic equivalence at the landscape scale for freshwater wetland mitigation. *Ecol. Appl.* **6**, 57–68 (1996).

42. N. M. van Rooijen, W. de Keersmaecker, W. A. Ozinga, P. Coppin, S. M. Hennekens, J. H. J. Schaminée, B. Somers, O. Honnay, Plant species diversity mediates ecosystem stability of natural dune grasslands in response to drought. *Ecosystems* **18**, 1383–1394 (2015).

43. B. Rathcke, E. P. Lacey, Phenological patterns of terrestrial plants. *Annu. Rev. Ecol. Syst.* **16**, 179–214 (1985).

44. E. E. Cleland, J. M. Allen, T. M. Crimmins, J. A. Dunne, S. Pau, S. E. Travers, E. S. Zavaleta, E. M. Wolkovich, Phenological tracking enables positive species responses to climate change. *Ecology* **93**, 1765–1771 (2012).

45. H. Fu, G. Yuan, E. Jeppesen, D. Ge, D. Zou, Q. Lou, T. Dai, W. Li, J. Zhong, Z. Huang, Q. Liu, A. Wu, Multiple stabilizing pathways in wetland plant communities subjected to an elevation gradient. *Ecol. Indic.* **104**, 704–710 (2019).

46. G. L. Qin, G. Z. Du, Y. J. Luo, G. S. Dong, J. Y. Ma, A reexamination of the relationships among phenological complementarity, species diversity, and ecosystem function. *Bot. Bull. Acad. Sin.* **44**, 239–244 (2003).

47. M. H. H. Stevens, W. P. Carson, Phenological complementarity, species diversity, and ecosystem function. *Oikos* **92**, 291–296 (2001).

48. L. Zhao, G.-X. Yang, Z.-L. Liu, X.-P. Xin, Y.-J. Luo, G. Wang, Phenological complementarity does not enhance ecosystem production in undisturbed steppe community. *J. Integr. Plant Biol.* **49**, 582–587 (2007).

49. N. Pettorelli, M. Wegmann, A. Skidmore, S. Mücher, T. P. Dawson, M. Fernandez, R. Lucas, M. E. Schaepman, T. Wang, B. O'Connor, R. H. G. Jongman, P. Kempeneers, R. Sonnenschein, A. K. Leidner, M. Böhm, K. S. He, H. Nagendra, G. Dubois, T. Fatoyinbo, M. C. Hansen, M. Paganini, H. M. de Klerk, G. P. Asner, J. T. Kerr, A. B. Estes, D. S. Schmeller, U. Heiden, D. Rocchini, H. M. Pereira, E. Turak, N. Fernandez, A. Lausch, M. A. Cho, D. Alcaraz-Segura, M. A. McGeoch, W.

Turner, A. Mueller, V. St-Louis, J. Penner, P. Vihervaara, A. Belward, B. Reyers, G. N. Geller, Framing the concept of satellite remote sensing essential biodiversity variables: Challenges and future directions. *Remote. Sens. Ecol. Conserv.* **2**, 122–131 (2016).

50. T. F. Keenan, I. Baker, A. Barr, P. Ciais, K. Davis, M. Dietze, D. Dragoni, C. M. Gough, R. Grant, D. Hollinger, K. Hufkens, B. Poulter, H. McCaughey, B. Raczka, Y. Ryu, K. Schaefer, H. Tian, H. Verbeeck, M. Zhao, A. D. Richardson, Terrestrial biosphere model performance for inter-annual variability of land-atmosphere CO_2 exchange. *Glob. Chang. Biol.* **18**, 1971–1987 (2012).

51. N. Gorelick, M. Hancher, M. Dixon, S. Ilyushchenko, D. Thau, R. Moore, Google Earth Engine: Planetary-scale geospatial analysis for everyone. *Remote Sens. Environ.* **202**, 18–27 (2017).

52. P. H. C. Eilers, A perfect smoother. *Anal. Chem.* **75**, 3631–3636 (2003).

53. E. T. Whittaker, On a new method of graduation. *Proc. Edinburgh. Math. Soc.* **41**, 63–75 (1922).

54. J. K. Ord, A. Getis, Local spatial autocorrelation statistics: Distributional issues and an application. *Geogr. Anal.* **27**, 286–306 (1995).

55. N. Clinton, L. Yu, H. Fu, C. He, P. Gong, Global-scale associations of vegetation phenology with rainfall and temperature at a high spatio-temporal resolution. *Remote Sens.* **6**, 7320–7338 (2014).

56. J. A. Dunne, J. Harte, K. J. Taylor, Subapline meadow flowering phenology responses to climate change: Integrating experimental and gradient methods. *Ecol. Monogr.* **73**, 69–86 (2003).

57. A. Schuster, On the investigation of hidden periodicities with application to a supposed 26 day period of meteorological phenomena. *J. Geophys. Res.* **3**, 13 (1898).

58. P. Welch, The use of fast Fourier transform for the estimation of power spectra: A method based on time averaging over short, modified periodograms. *IEEE Trans. Audio Electroacoust.* **15**, 70–73 (1967).

59. E. R. Bush, K. A. Abernethy, K. Jeffery, C. Tutin, L. White, E. Dimoto, J. Dikangadissi, A. S. Jump, N. Bunnefeld, Fourier analysis to detect phenological cycles using long-term tropical field data and simulations. *Methods Ecol. Evol.* **8**, 530–540 (2017).

60. X. Zhu, M. Raquel, V. Aryadoust, in *Quantitative Data Analysis for Language Assessment Volume II* (Routledge, 2019), pp. 101–126.

61. I. Forbrich, A. E. Giblin, Marsh-atmosphere CO_2 exchange in a New England salt marsh. *Eur. J. Vasc. Endovasc. Surg.* **120**, 1825–1838 (2015).

62. D. Garcia, Robust smoothing of gridded data in one and higher dimensions with missing values. *Comput. Stat. Data Anal.* **54**, 1167–1178 (2010).

63. US EPA, National Wetland Condition Assessment 2016: Field Operations Manual (EPA-843-R-15-007, US EPA, 2015).

64. N. P. Robinson, B. W. Allred, W. K. Smith, M. O. Jones, A. Moreno, T. A. Erickson, D. E. Naugle, S. W. Running, Terrestrial primary production for the conterminous United States derived from Landsat 30 m and MODIS 250 m. *Remote Sens. Ecol. Conserv.* **4**, 264–280 (2018).
